# Does climate help modeling COVID-19 risk and to what extent?

**DOI:** 10.1371/journal.pone.0273078

**Published:** 2022-09-07

**Authors:** Giovanni Scabbia, Antonio Sanfilippo, Annamaria Mazzoni, Dunia Bachour, Daniel Perez-Astudillo, Veronica Bermudez, Etienne Wey, Mathilde Marchand-Lasserre, Laurent Saboret

**Affiliations:** 1 Qatar Environment and Energy Research Institute, Hamad Bin Khalifa University – Qatar Foundation, Doha, Qatar; 2 Transvalor S.A, Sophia Antipolis, France; Valahia University of Targoviste, ROMANIA

## Abstract

A growing number of studies suggest that climate may impact the spread of COVID-19. This hypothesis is supported by data from similar viral contagions, such as SARS and the 1918 Flu Pandemic, and corroborated by US influenza data. However, the extent to which climate may affect COVID-19 transmission rates and help modeling COVID-19 risk is still not well understood. This study demonstrates that such an understanding is attainable through the development of regression models that verify how climate contributes to modeling COVID-19 transmission, and the use of feature importance techniques that assess the relative weight of meteorological variables compared to epidemiological, socioeconomic, environmental, and global health factors. The ensuing results show that meteorological factors play a key role in regression models of COVID-19 risk, with ultraviolet radiation (UV) as the main driver. These results are corroborated by statistical correlation analyses and a panel data fixed-effect model confirming that UV radiation coefficients are significantly negatively correlated with COVID-19 transmission rates.

## Introduction

The COVID-19 pandemic has triggered an extensive amount of research across different fields, from epidemiology to the social sciences. One of the issues under investigation pertains to the heterogeneous character of COVID-19 diffusion across diverse geographic areas. The debate on this matter has given rise to two main approaches. The first is primarily based on the epidemiological explanation of contagion dynamics in terms of factors such as frequency and intensity of physical contact among people and their viral charge. According to this approach, the only variables capturing the viral spread are contagion dynamics factors and the ability of policymakers to reduce the contagion rate by restricting population mobility. The second approach evaluates the possibility that contagion dynamics result from multiple factors, including meteorological and environmental variables.

Since the early stages of the COVID-19 pandemic, climate has provided an important reference point to explain the spread of the virus. Just three months after the first outbreak in Wuhan, China, Bukhari and Jameel [1] reported that 90% of COVID-19 cases were recorded through 22 March 2020 in world areas with colder and less humid late winters and early springs (temperatures of 3–17°C, and absolute humidity of 4–9 g/m^3^). As recognized by the authors, these early data were likely to be biased by minimal testing per capita in tropical countries. After over two years into the pandemic, 226 countries across the globe have reported a total of over 500 million confirmed COVID-19 cases [2]. We now have a clearer picture of the global distribution of this disease. There are numerous examples indicating that the cooler season in the northern hemisphere may have favored the spread of the disease, while warmer and more humid weather in late spring and summer has seen a substantial and rapid decline in transmission numbers, once the different containment strategies adopted worldwide are taken into consideration [3, 4].

Prior work on the betacoronavirus genus shows that viruses similar to SARS-CoV-2, such as HCoV-HKU1 and HCoV-OC43, also display seasonal peak trends [5]. The spread of previous coronavirus epidemics, such as SARS-related and the Middle East respiratory syndrome (MERS)-related coronaviruses, have also displayed correlations with seasonal weather changes [6–9]. More specifically, outbreaks of respiratory virus infections are commonly associated with seasonality, with peaks during the winter months. Data from the 1918–19 Flu Pandemic support this correlation. Peak infection/mortality during the 1918–19 Flu Pandemic occurred in the winter months and waned as solar radiation and absolute humidity increased from late March onward. The resurgence of mortality in the winter of 1919 and its decline with the arrival of spring confirm this trend. These observations are corroborated by US influenza data relative to the last decade where the percentage of patient visits for influenza-like illness consistently grows in the winter months.

Regression analyses correlating meteorological factors with the spread of SARS-CoV-2 have reported contrasting results. A number of studies have found a negative correlation between temperature and the spread of COVID-19 in China and the US [10–16], Italy [17, 18], Spain [19], Mexico [20], Brazil [21], Latin America and the Caribbean [22], and worldwide [23–27]. By contrast, Xie and Zhu [28] suggest a positive relationship between temperature and the spread of COVID-19 (up to 3°C), and city-level data from Oslo, Jakarta and, five Brazilian cities show a positive correlation between COVID-19 transmission and higher temperatures, and negative correlation with precipitations [29–31] which typically lead to lower temperatures. Studies on the impact of humidity have also given conflicting results with reports of positive correlation by Jiang et al. [10] for China, and the opposite in studies by Ward et al. [32] for Australia, Qi et al. [11] and Wang et al. [12] for China, Yin et al. [21] in Brazil, and Jüni et al. [33] and Aboura [34] worldwide. Several global analyses and reviews on the impact of climate on COVID-19 spread [35–39] have also reported conflicting results.

Other meteorological factors investigated with reference to COVID-19 transmission include wind and solar radiation. Most studies focusing on the impact of wind speed on the incidence of COVID-19 cases have found a negative correlation [40–42]. Studies focusing on solar radiation in Brazil [43], Australia [44], and worldwide [27, 45, 46] have found that high exposure to solar radiation including UV is negatively correlated with the occurrence of COVID-19 cases.

S1 Table provides a summary of the peer-reviewed research on the interrelationship between COVID-19 and meteorological/climatic factors discussed in this section. Overall, there is no conclusive evidence that climate plays a role in the spread of COVID-19 [47–50]. This is probably due to the fact that studies on the impact of climate on COVID-19 transmission have been piecemeal (e.g., limited to country or administrative division-level data), have only taken into account a few climatic parameters, and have not considered the impact of socioeconomic factor, as remarked in Mecenas et al. [4]. The present study addresses these impediments by investigating the contribution of meteorological factors to modeling COVID-19 transmission at the global scale with reference to reported COVID-19 cases in 196 countries over a 14-month period, using socioeconomic, environmental, and global health factors as control variables. The study employs three complementary approaches to measure the correlation between reported rates of COVID-19 transmission and selected meteorological, socioeconomic, environmental, and global health factors. The first approach is based on the statistical analysis of the rank correlation of every factor with the number of daily confirmed COVID-19 cases. Following is a feature importance analysis that uses Shapley Additive Explanations (SHAP) to elucidate the COVID-19 rate predictions of a regression model by computing the contribution of each feature in the model to the prediction task. The third and last approach is based on econometric analysis with panel data fixed-effect regression models. The three approaches supply independent analytic evidence of the relationship between confirmed COVID-19 cases and meteorological factors. Of particular interest is the complementarity between the machine learning analysis, which is intent on prediction, and the econometric analysis, which focuses on explanation. As discussed in the literature, the use of a hybrid approach where machine learning modeling is paired with econometric analysis can help address relative weaknesses in the two methods by leveraging relative strengths. Further details are provided in the Methodology subsection below.

## Materials and methods

### Data sources and pre-processing

The data used in this study include epidemiological, socioeconomic, environmental, global health indicators, and meteorological variables–see S2 Table. All population-related variables are converted to percentages of the total population per country. Parameters that behave as time-invariant variables during the period of focus for this study, e.g., socioeconomic variables, are used as control variables.

Epidemiological data on the cumulative number of confirmed COVID-19 cases were retrieved for the period from 23 January 2020 to 21 March 2021 from two main sources: the data repository from the Johns Hopkins Center for Systems Science and Engineering [51], and the Corona Data Scraper online data service that retrieves COVID-19 Coronavirus cases data from verified sources worldwide and adds population data on a daily basis (coronadatascraper.com). Data from these two sources were merged to create the first dataset. In addition to country-level records, data at the regional or state level were included when available. We derived the number of daily registered COVID-19 cases by differencing entries in the initial dataset. Data points characterized by a modified Z-score > 3.5 and with values above the third quartile or below the first quartile and 1.5 times greater than the interquartile range were discarded as outliers together with other inconsistent data points (e.g., negative values) [52]. From the daily values, we retrieved 3-day and 7-day moving averages as features to incorporate an auto-regressive component in the learning model. Only records reporting more than 10 daily cases were included in the analysis. The time of exposure to the pandemic for each country was also calculated as the cumulative temporal distance from the first registered case in the country. We selected data through March 2021, when most countries began their vaccination campaigns, to avoid including vaccination as an additional impact on COVID-19 transmission rates. Such inclusion would have created discrepancies with earlier data and introduced inconsistencies emerging from the adoption of diverse vaccination strategies worldwide.

We used several socioeconomic time-invariant data sources including demographic information, technology adoption rates, and Gross Domestic Product per-capita (GDPP) as control factors in the cross-sectional fixed effect analysis. Demographic, population density, and population age data were derived from the 2019 Population Division dataset compiled by the Department of Economic and Social Affairs of the United Nation (UNDESA) [53] and the World Bank indicators database [54]. Information for geographical locations not included in the UNDESA dataset was retrieved online from national official sources. Rates of internet users, subscribers to mobile telephony services, and the number of secure Internet servers were retrieved from the World Bank indicators database. These technology adoption variables are used as proxies for the capacity of different countries to provide smart-work environments under lockdown, create awareness and keep the population updated about the development of the pandemic, and support effective contact tracing applications. GDPP data at constant price purchasing-power-parity were sourced from the International Monetary Fund’s World Economic Outlook Database [55].

Environmental indicators retrieved from the World Bank database include population-weighted exposure to ambient PM2.5 pollution, carbon dioxide, methane, nitrous oxide emissions, and greenhouse gas emissions. These time-invariant variables were used as control indicators of the degree of pollution in each country, on the assumption that long-term exposure to pollutants may increase the risk of contracting COVID-19 given that outdoor air pollution has been positively correlated with respiratory diseases.

Health indicators included time-invariant variables such as the general Global Health Security (GHS) index, the GHS detect and prevent scores, diabetes prevalence, and the number of hospital beds for both acute and chronic care. GHS provides a country-level score of health security and was used as a proxy variable for a country’s capability to prevent and mitigate infectious diseases. For the purpose of this study, only the “detect” and “prevent” GHS categories were used, which focus on a country’s readiness to promptly identify, report, and anticipate disease outbreaks of potential international concern [56]. Health indicators relative to diabetes prevalence and the number of hospital beds for both acute and chronic care were retrieved from the World Bank database. These variables serve as proxies for population health status and public health preparedness.

Differences in intervention responses by governments to mitigate the pandemic were accounted through a variety of indicators from the Oxford COVID-19 Government Response Tracker (OxCGRT) project [57, 58]. The OxCGRT variables include policy information on school closures (C1), workplace closures (C2), cancellation of public events (C3), restriction on gatherings (C4), closure of public transports (C5), lockdowns (C6), and restriction on internal (C7) and international (C8) movements and travels. In addition, the OxCGRT database provides health system policy data on the presence of public information campaigns (H1), testing policy (H2), contact tracing (H3), and facial covering policies (H6). Finally, we used two more variables that are calculated as a weighted aggregation of the single C and H indices: the stringency and containment & health indices. The first variable reflects the strictness of “lockdown style” policies that primarily restrict people’s behavior. The second combines “lockdown” restrictions with measures such as testing policy and contact tracing, short-term investment in healthcare, and investments in vaccines. OxCGRT data are provided at a country-level, with subnational data available for Canadian provinces, US states, and UK regions (New England, Northern Ireland, Scotland, and Wales).

Meteorological variables were obtained from two main sources: the Modern-Era Retrospective analysis for Research and Applications, Version 2 (MERRA-2), a gridded reanalysis dataset produced by NASA’s Global Modeling and Assimilation Office (GMAO), and the Copernicus Atmosphere Monitoring Service (CAMS) with specific reference to the McClear Clear-Sky Irradiation model. The MERRA-2 data include daily averages of the original hourly data at a spatial resolution of approximately 50 km, temperature in °C, relative humidity at 2 m above ground in %, short-wave solar irradiation (total of the day) in Wh/m^2^, pressure at 2 m from ground level (station pressure) in hPa, wind speed at 10 m above ground in m/s, and rainfall in mm. Temperature is combined with relative humidity to derive measures of absolute humidity in g/m^3^ [59]. Data obtained from CAMS include downward UV Radiation at the Surface (UV) in J/m^2^, and Particulate Matter (PM) concentrations (PM2.5 and PM10) for 3-hour periods at a spatial resolution of 40 km. UV exposure can have a sterilizing effect [60] and ultraviolet B light (UVB), which is present in small amounts in natural sunlight, is known to rapidly inactivate SARS-CoV-2 on surfaces [61]. Data on particulate matter [62], originally in kg/m^3^, is converted to micrograms/m^3^ and it can provide preliminary evidence on the relation between air quality and the chronicity of exposure to the viral infection. Coccia [63] and Bloise and Tancioni [64] both suggest that air pollution may have accelerated the transmission rate of COVID-19 in northern Italy, even though the viability of infectious viruses embedded in suspended aerosol particles is still under debate [65].

Streaming access to MERRA-2 and CAMS was provided by Transvalor S.A. For each location considered in the study, we derived the geographical centroids of the country’s most populous cities [66]. For country-level locations, we considered the ten most populated cities, while for admin-level locations (sub-country, i.e., states, territories, provinces, cantons, etc. as appropriate per country) we considered the five most populous cities. We used the corresponding latitude and longitude coordinates of each city to query the climatic information from MERRA-2 and CAMS through Transvalor’s SoDa data service (http://www.soda-pro.com/) and we finally derived the resulting time-variant meteorological data by averaging across the different cities for each location.

After merging all the various sources, the resulting dataset includes data on 196 countries covering 96% of the world population and 97.6% of worldwide confirmed COVID-19 cases (123,491,126 –at the period of the study). Data for 28 of these countries are detailed in the dataset at a state or regional level (i.e., admin-level) for the available periods (see S1 File for a detailed list). We only consider country-level epidemiological data for the remaining 152 countries, even though admin-level data are available from coronadatascraper.com, in order to keep a certain level of minimum comparison between the locations under study in terms of overall population size. S2 and S3 Tables provide detailed information on the variables used for the study and their descriptive statistics.

The COVID-19 mean incubation period, defined as the time period ranging between exposure to the virus and the onset of the illness, is estimated by WHO at 5–6 days (median 5.1 days, 95% Confidence Interval (CI): 4.5 to 5.8 days) [67]. According to Lauer et al. [67], 97.5% of those who develop symptoms will do so within 11.5 days (CI: 8.2 to 15.6 days) from the day of infection. Moreover, the results of a COVID-19 PCR test have been known to take up to an average of 3–4 days (3.6 days according to Cereda et al. [68]), particularly during the initial months of the pandemic. For these reasons, the number of new COVID-19 cases that are officially announced each day corresponds to a time window of infection that spans from a few days up to potentially two weeks earlier. To account for this timeframe uncertainty and test the robustness of our results, the analysis is carried out with moving averages for both the time-variant meteorological and the policy variables of different duration: 5, 7, 10, 12, and 14 days (minimum length of 3 days). In reporting results, the number of days determining the moving average of time-variant variables is encoded as either a number suffix (e.g., Temperature_7), or a suffix variable indexed to a specific numeric value (e.g., Temperature_T, … T = 7).

### Limitations and assumptions

As for other data-driven studies on COVID-19 transmission, the present analysis relies on records whose quality varies across sources, due to heterogeneous collection and reporting practices worldwide. Data quality, extensiveness, and uniformity are therefore subject to a certain degree of uncertainty. Moreover, reports of confirmed COVID-19 cases tend to underestimate the actual number of infections because of asymptomatic patients and undetected COVID-19 deaths. For ease of purpose, we will assume that the number of confirmed COVID-19 cases is monotonically related to the true number of infections, recognizing that this is a simplification that may limit the significance of this study’s results.

An additional but less impactful limitation emerges from the normalization of meteorological variables across different points within each area of interest where exposure to the virus has occurred. As previously described, weather conditions have been averaged across the ten most populous cities for each country-level location and the five most populated cities for the admin-level locations. This approach should provide a good approximation of the overall weather conditions under which the viral spread has occurred. We reason that population density may have played a significant role in the spread of COVID-19. Furthermore, it is also likely that the majority of testing was carried out in major urban areas. Finally, selecting up to ten country-level and five admin-level cities can be expected to evenly spread the sampling across the most populated areas, as such an approach provides an average coverage of 75% or more of the entire population for most of the locations considered (S1 Fig).

### Methodology

The method used in this study combines three approaches to capture dependencies between confirmed COVID-19 cases and climate factors (see S2 Fig for a visual diagram summarizing the approach):

- A statistical analysis based on Spearman’s and Kendall’s correlation coefficients
- A machine learning model based on the Gradient Boosted Regression Tree algorithm paired with the Tree SHAP algorithm to perform feature importance analysis
- An econometric model based on fixed effect panel regression analysis.

The use of three distinct approaches is intended to provide independent analytic evidence. Of particular interest is the complementarity between machine learning and econometric approaches, where the first is intent on prediction while the latter focuses on explanation. As discussed in the literature, the use of a hybrid approach where machine learning modeling is paired with econometric analysis can help address relative weaknesses in the two methods by leveraging relative strengths [69–73]. For example, machine learning is better equipped to take advantage of structural heterogeneity in training data to make short-term predictions, whereas econometric methods are better at capturing long-term trends [73]. We can therefore expect that the results of machine learning and econometric analysis are not always going to coincide [73]. This lack of overlap points to the areas of relative improvement that can be obtained through a functional integration of the two methods. While appealing, such integration is challenging and largely remains a goal to be achieved, for which a better understanding of the differences and relative strengths/weaknesses is required [71]. In this regard, the present study contributes to advancing our understanding of the specific complementarities between machine learning and econometrics in a new domain of inquiry. The statistical analysis in turn provides the baseline due to its more basic analytic capacity in dealing with non-stationary processes compared to machine learning and accounting for long terms trends compared to econometric analysis.

Finally, the validity of the approach adopted in this study is corroborated by the research framework for linking environmental and weather factors to the incidence of COVID-19 proposed in a recent study published by Zaitchik et al. [74].

### Statistical analysis

The statistical dependency of confirmed COVID-19 cases from environmental and meteorological regressors is first performed by calculating and comparing Spearman and Kendall rank-order correlation coefficients. These coefficients provide a nonparametric measure of the monotonicity (i.e. strength and direction) of the relation between the number of confirmed COVID-19 cases (the output/dependent variable) and the input environmental and meteorological variables. Unlike the Pearson correlation, the Spearman and Kendall rank-order correlations do not carry any assumptions about the normal distribution of the data and the linearity between the variables. The statistical significance of the association between input and output variables is determined using the two-sided p-value in order to measure both decreasing and increasing departures from the null hypothesis. Spearman’s and Kendall’s correlation coefficient values (*ρ* and *τ*) can range from +1 to -1. The sign of the coefficient indicates the direction of the association of ranks (+ positive,—negative), while its absolute value expresses magnitude. The closer the coefficient is to zero, the weaker the association between the ranks: an absolute value between 0.5 and 1 is considered to provide a strong correlation, 0.3 to 0.5 a moderate correlation, 0.1 to 0.3 a weak correlation, and <0.1 no correlation.

### Machine learning modeling

The aim of the machine learning analysis is to assess the relative feature impact of factors contributing to COVID-19. Feature impact is computed by applying the Tree SHAP algorithm to a Gradient Boosted Regression Tree (GBRT) model. GBRT is an additive stochastic model that combines multiple sequentially connected weak learners (regression trees) in a way that each new learner fits the residuals from the previous step to optimize the overall predictive performance [75]. The resulting model can describe multiple nonlinear interactions and partial dependency with sufficient flexibility, remarkably high predictive accuracy, and robustness to missing data and outliers.

The study uses the open source xgboost Python library which offers a highly efficient, flexible, and portable implementation of GBRT [76]. The xgboost algorithm provides several ways to control overfitting, i.e., when the model fits the training data so closely that it fails to provide useful predictions when applied to new data. The first is to constrain the maximum depth of individual trees used in the boosting process to modulate the degree of feature interactions that the model can fit. The second is to control the number of samples that each tree leaf can contain to avoid forming imbalanced leaves that have a single or too few data points. The third and most important way is to control the learning rate. Overfitting is also reduced through the use of randomization into the tree building process by subsampling the training set before deriving each tree, and subsampling features before searching for the best node split. Finally, xgboost provides a parameter that enables model regularization using "across trees" information.

As a first step, we optimized the model hyperparameters using a grid-search method combined with a cross-validation approach specifically designed for this study. We first selected only the data records of geographical locations that presented at least 90 data points (about 3 months’ worth of data) filtering out about 12,000 observations from a total of 77,300. This strategy is intended to select a time window size that presents sufficient seasonal variation for each location. We then randomly selected 24 locations to be designated as a test set to measure the final unbiased performance of the optimized model. Each location in the filtered dataset has on average 273 data points, thus resulting in an overall test set size of ~6,500 observations (about 10% of the starting dataset) and a training set of 60,000 observations. We used a grid-search approach to find the best model hyperparameters validated on a further 10% split share of the training set. This validation set was derived by randomly selecting 22 locations for a total of ~6,000 observations, thus leaving 54,000 data points for training the model with the specific parameters under validation. For each parameter, we repeated this procedure 5 times to assure that the resulting validation error score (in terms of symmetric mean absolute percentage error—SMAPE) would converge. During each reiteration, we re-selected a new random set of 22 locations from the overall training set as the new validation set. This step led to a measurable improvement in the prediction accuracy over the same algorithm initialized with the default hyperparameters values (base model).

**Fig 1:**
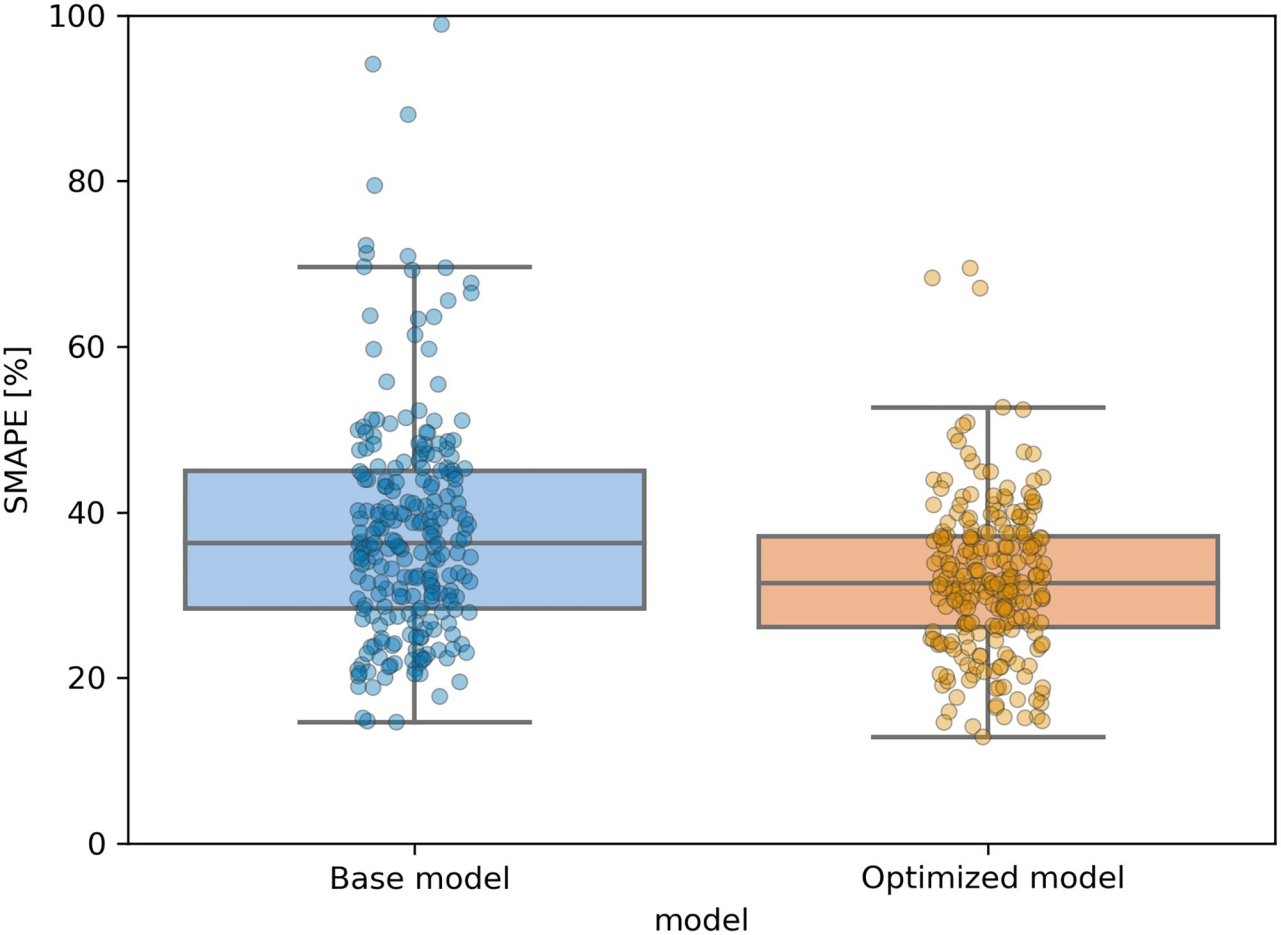
Accuracy of the based model (left, blue) compared to the optimized model (right, orange) on the test set. We consider only locations with more than 90 data records and records with more than 10 cases per day.

Fig 1 shows the performance gain in terms of lower SMAPE error of the optimized model compared to the base model computed on the first randomized test set, which was never seen by both models. On average this optimization procedure results in a ~5–10% lower SMAPE. Table 1 summarizes the set of hyperparameters leading to the best evaluation results. Table 2 reports the training, validation, and test performance of the GBRT model (default and optimized) compared to two deterministic baselines: the prediction obtained by using a 7-day moving average, and a persistence model, where the value of the predicted dependent variable is assumed to be the same as the previous day. For comparison, we also report the performance of other regression models such as Lasso, Elastic Net, and Random Forest after a cross-validated tuning of their hyperparameters. The optimized GBRT model outperforms all other models on the test set with a mean SMAPE error of 7.5% lower than the base model, 2.4% smaller than the Random Forest model, 11% better than the Lasso model, and 24% lower than the Elastic Net model. High error values for Lasso and Elastic Net are likely related to their lower model complexity that prevents the proper learning of data interrelationships. Random Forest produces a comparable accuracy to xgboost, but it shows overfitting on the train set. Finally, when compared to the two deterministic baselines the tuned xgboost model produces predictions on the test set that are 20.5% and 4% more accurate than the 1-week moving average and persistence models respectively.

**Table 1:** Xgboost hyperparameter tuning result.

Hyperparameter	Tuning range	Best value
Learning rate (eta)	0.001–0.3	0.1
Maximum depth	3–10	10
Minimum sum of instance weight (hessian)	1–10	7–8*
Gamma	0–0.4	0.2*
Subsample ratio of the training instances	0.5–1	1*
Subsample ratio of columns when constructing each tree	0.3–1	1*
Lambda	1	1
Alpha	0	0
Number of boosting rounds (validated)		~30
Learning objective function (regression)		Squared error
Custom evaluation metric (for training and validation)		SMAPE
Early stopping rounds		10

*Irrelevant to the model performance.

**Table 2:** Regression modeling performance comparison.

Model	Mean SMAPE		
	Train	Validation	Test
GBRT (with hyperparameter optimization)	28.3%	30.6%	**29.0%**
GBRT (no optimization)	33.5%	-	**36.5%**
Lasso*	45.7%	41.9%	**40.1%**
Elastic Net*	60.2%	60.5%	**53.3%**
Random Forest*	11.9%	31.2%	**31.4%**
1-week moving average	-	-	**49.5%**
Persistence (previous day)	-	-	**34.9%**

*With hyperparameter optimization

**Fig 2:**
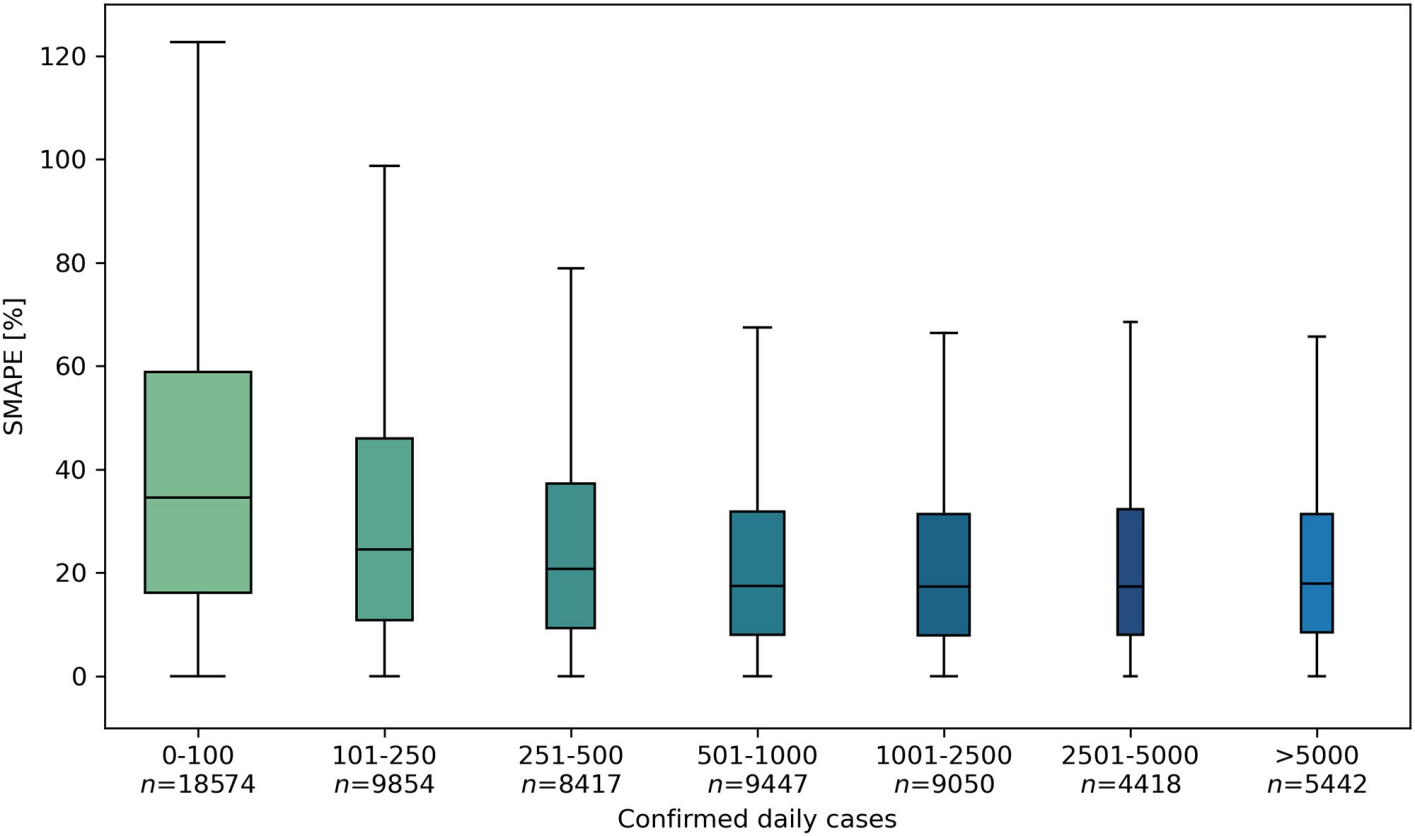
Boxplot of the SMAPE distribution as a function of intervals of number of COVID-19 daily cases.

Fig 2 shows model accuracy distribution as a function of the daily cases grouped in different intervals for all the locations considered in this study. The width of each boxplot is proportional to the number of observations included in the specific range (n), which is also reported below the label of each interval. The cross-sectional median error of the model decreases with the increase of the values of the dependent variable, going from 35.1% when daily cases are < 100 down to 17.5% with daily cases >500.

**Fig 3:**
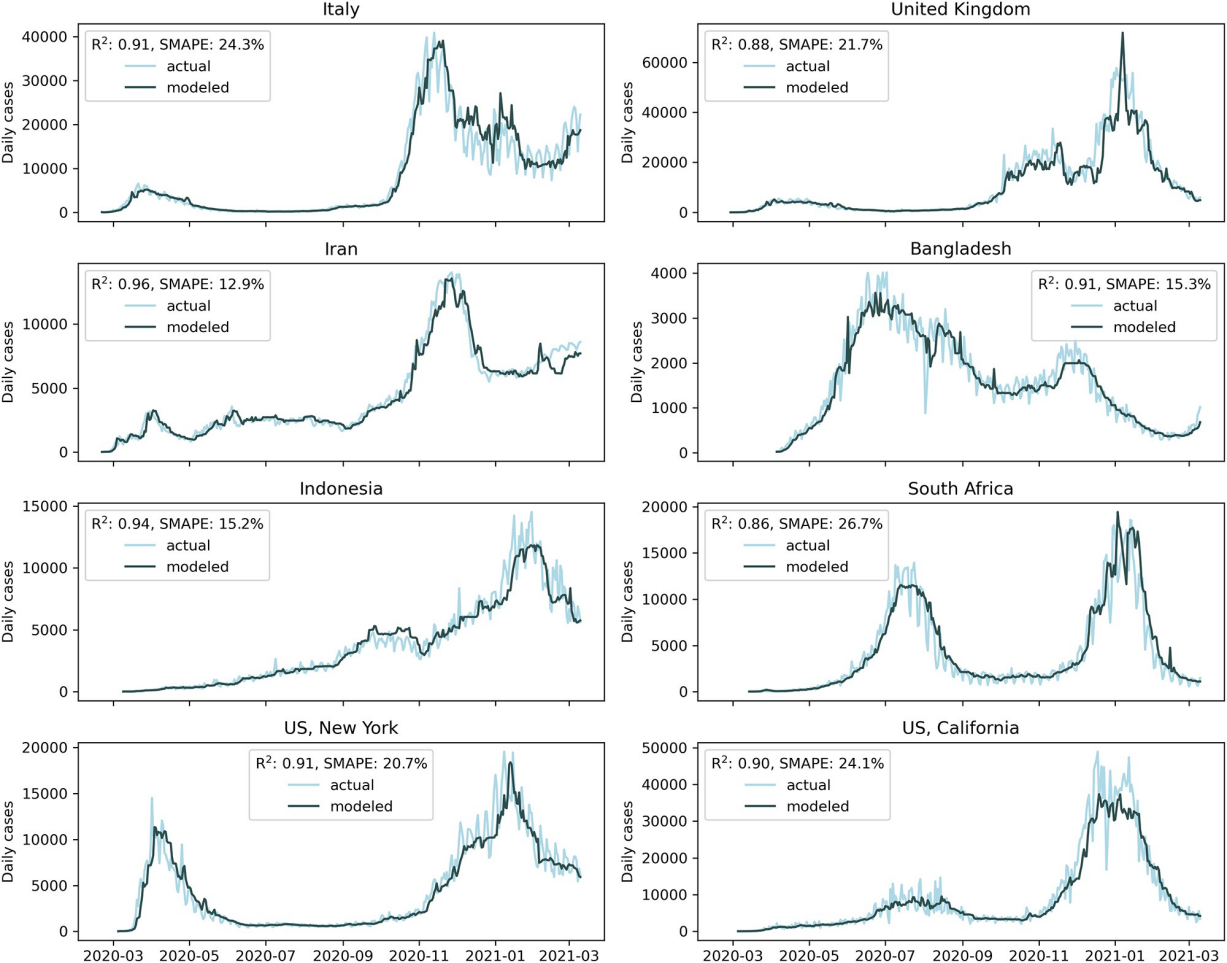
Examples of modeling performance for the optimized GBRT model.

To obtain a more complete estimate of model accuracy we tested the performance of the optimized GBRT model for each distinct location. We selected areas from different climatic zones that presented high numbers of daily cases during one or several contagion waves throughout the year. The results of this evaluation reveal error rates ranging from 12.9% to 26.8%, as shown in Fig 3.

Once the GBRT model is trained, the relative ranking of model parameters is obtained through the SHAP method. Tree SHAP is an algorithm that computes SHAP values for Decision Trees models such as GBRT. SHAP (SHapley Additive exPlanation) [77, 78] uses a game theoretic approach to explain the prediction for each instance as a sum of contributions from its individual feature values. This type of analysis does not identify causal correlation, but it is still a useful metric to capture relative feature importance.

### Econometric analysis

The econometric analysis of the association between confirmed COVID-19 infections and climatic factors is carried out using the multivariate equation in (1) which implements a panel data approach based on a fixed-effects model [79]. In this equation, the dependent variable *ln*_*daily*_*cases*_*i*,*t*_ expresses the number of daily cases of COVID-19 cases on a logarithmic scale for location *i* and time index *t*. We use the log-transformed version of the dependent variable on the assumption that by doing so the variable becomes log-normal conditional on all the covariates and thus allows us to limit the heteroscedasticity of the estimated residuals. *β*_0_ is the regression intercept, while *β*_*n*_ represents the regression slope coefficient of each respective regressor. We include a vector of cross-sectional unit fixed effects *c*_*i*_ to account for all time-invariant factors across a location that affect the local growth rate of infections, such as differences in demographics, socioeconomic status, culture, and health systems. This is an important feature since it allows to partial out heterogeneous omitted factors that might be correlated with the dynamics of contagion and the daily cases. We also include a vector of (daily) time fixed effects *λ*_*t*_ to absorb the autoregressive component specific to the COVID-19 spread growth and to account for the presence of any potential seasonal bias. Since the effect of variables that behave as time-invariant factors for the period of focus (e.g., socioeconomic, environmental, and some global health indicator variables) would be absorbed in the intercept for collinearity due to the use of time fixed effect regression, these variables were omitted from the analysis. Finally, we cluster the standard errors *u*_*i*,*t*_ at the entity-level to account for error correlation within each location.


$$ln\_daily\_cases_{i,t}=\beta_{0}+\overset{N}{\underset{n=1}{\sum }}\;\beta_{n}\;Explanatory\_variables_{n,i,t}+c_{i}+\lambda_{t}+u_{i,t}$$ (1)

## Results

We designed a cross-national data-oriented study using global records of confirmed daily cases of COVID-19 to examine the association between the pandemic growth and climatic conditions, using several socioeconomic, environmental, and global health factors as control variables. We first apply Spearman’s and Kendall’s rank-order correlation analysis to the selected data to derive a first estimate of the statistical relationship between each explanatory variable and COVID-19 transmission at each geographic location under study. We then use machine learning techniques to model and understand the relative importance that climatic and control variables have on the spread of COVID-19. We finally use panel data econometric analysis to estimate the impact of climatic conditions on COVID-19 daily rates and test the efficacy of different containment policies.

### Statistical correlation analysis

**Fig 4:**
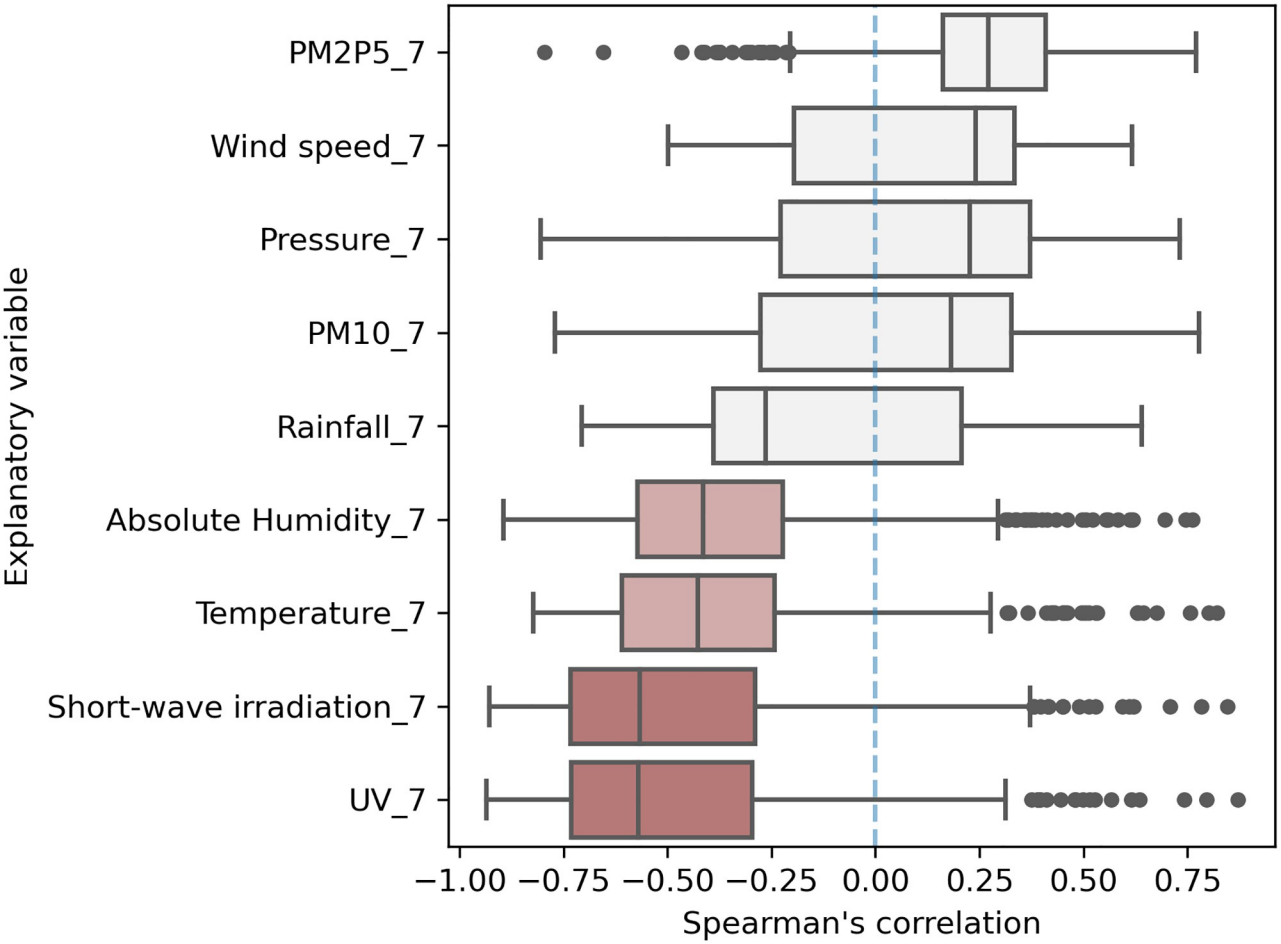
Spearman’s coefficient.

Fig 4 provides the distribution of Spearman’s rank correlation coefficients (*ρ*) that model the dependency of COVID-19 daily rates on the environmental and meteorological explanatory variables used in this study. Correlation coefficients are calculated for each location and time series. Results are clustered based on the specific location and consider only geographical areas with at least 90 data records (at least 3 months of data). Correlation coefficients greater than 1.5 IQR (interquartile range) that are below the first quartile or above the third quartile are considered outliers and reported in Fig 4 as scatter points. Only locations that showed statistical significance (P < 0.01) were considered and displayed in the descriptive analysis reported in Fig 4 (see S2 and S3 Tables for variable description and statistics).

**Fig 5:**
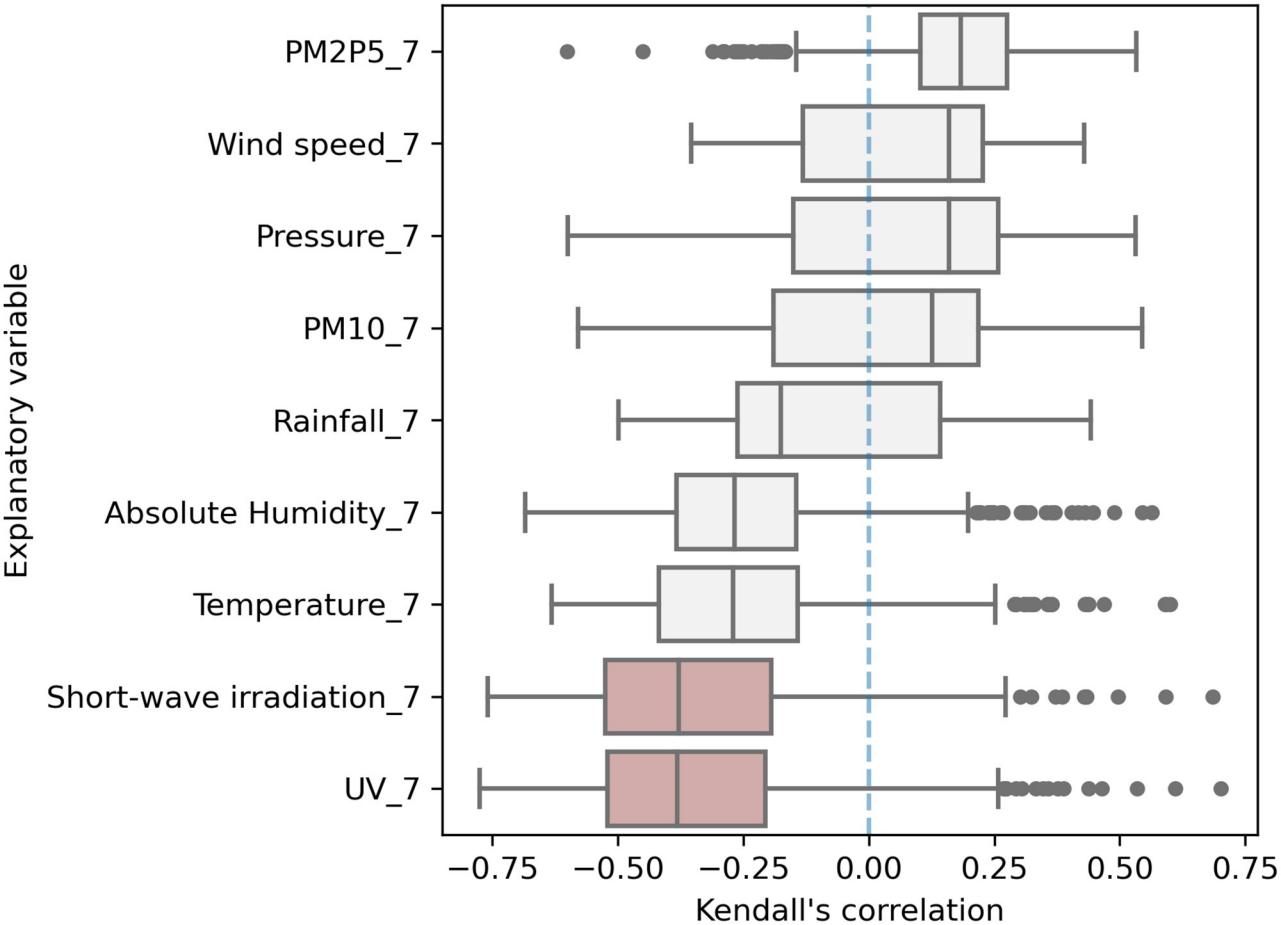
Kendall’s coefficient.

The Spearman’s rank correlation results suggest that solar irradiation and UV emission are strongly negatively correlated with COVID-19 spread (median *ρ* equal to -0.55 and -0.56, respectively). Temperature and absolute humidity also show a negative correlation, but with a weaker amplitude (at median values of -0.42 and 0.39, respectively; moderately correlated). The other meteorological and air-quality factors do not show a significant association with COVID-19 transmission (low |*ρ*| and large IQR: high standard deviation). PM2.5 concentrations and pressure register a positively weak correlation. All other variables present a weakly negative correlation. These results are corroborated by Kendall’s rank correlation analysis shown in Fig 5.

### Feature importance analysis

Relative feature predictive value is computed by applying the Tree SHAP (SHapley Additive explanation for tree-based machine learning models) algorithm to a Gradient Boosted Regression Tree (GBRT) model (see Methodology section for details). The GBRT model was trained on the whole dataset including all geographical locations independently from the number of its data records. Epidemiological, meteorological, socioeconomic, environmental, and global health indicator indices were used as explanatory variables, while the number of Covid-19 daily cases served as the dependent variable. See the Methodology section for a detailed definition of the variables. S2 and S3 Tables present a brief description of the variables included in the study and their summary statistics.

**Fig 6:**
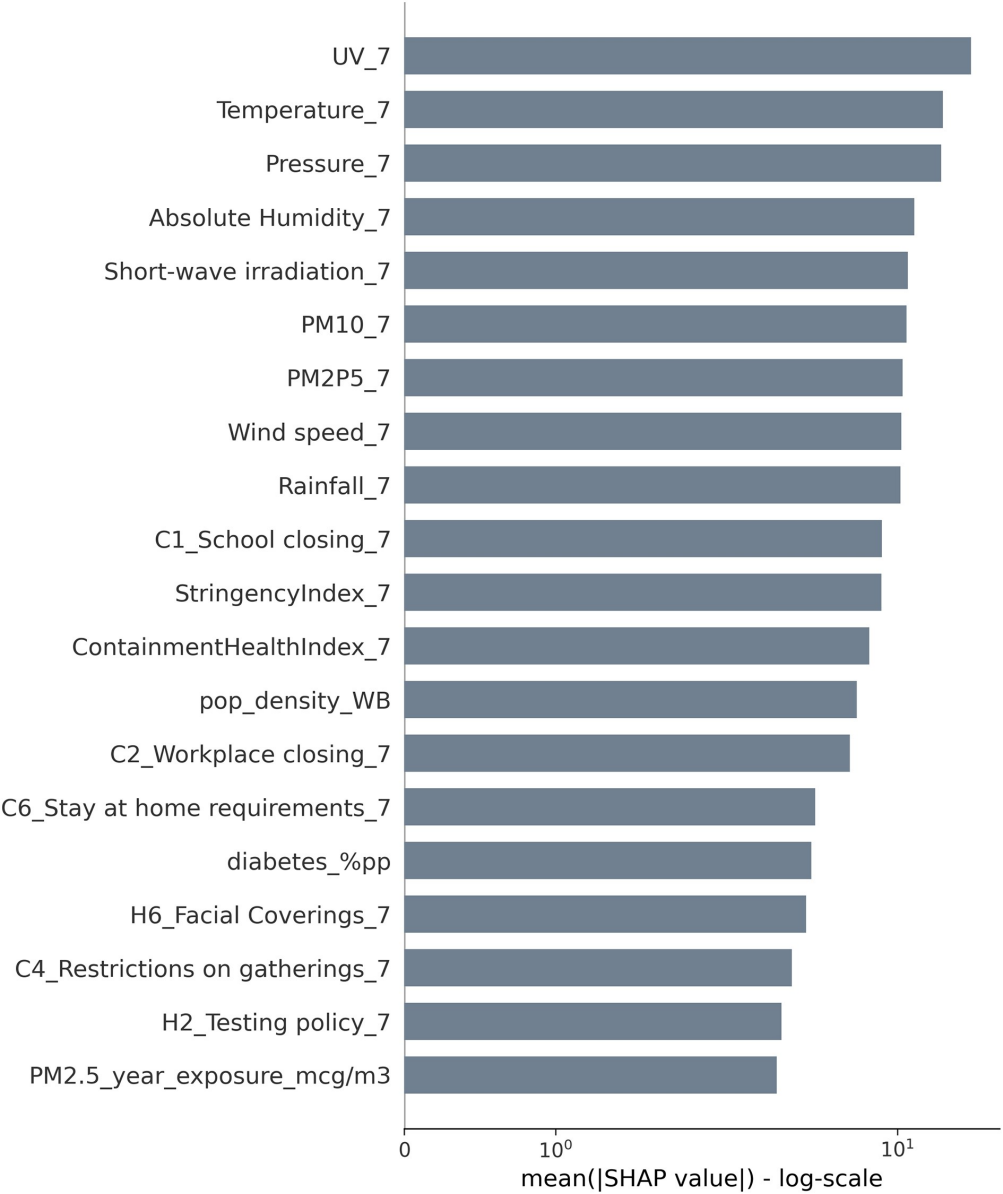
Feature importance summary plot. Mean absolute SHAP value (in log scale) of each variable showing the average impact on the model output magnitude.

**Fig 7:**
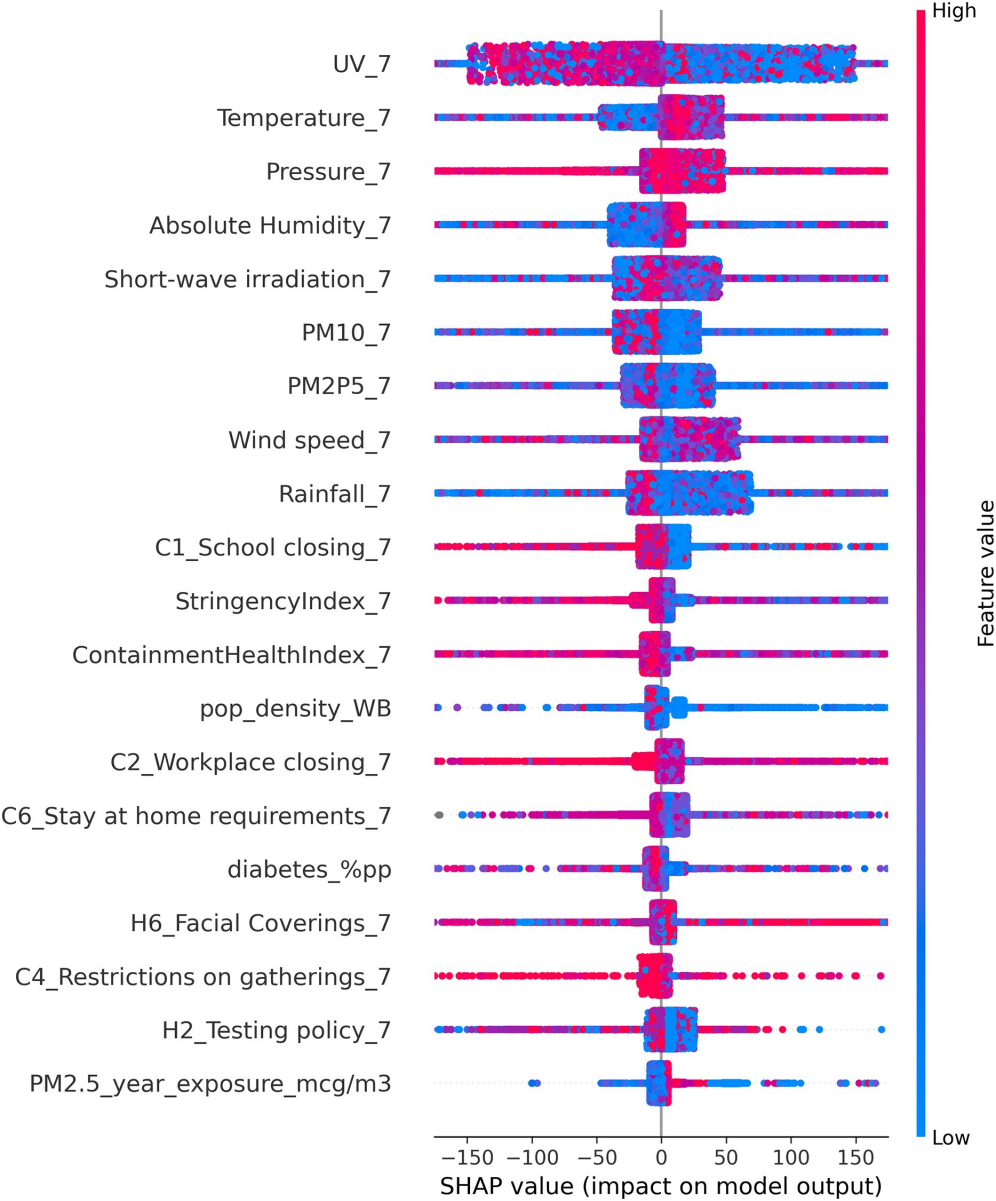
Feature impact scatter plot. SHAP value of each variable for all the single observations as a function of their relative value. The color transition on the vertical axis indicates value strength (red/high to blue/low).

The results of feature importance analysis suggest that climate plays a meaningful role in modulating the dynamics of the COVID-19 pandemic, as shown in Fig 6 where feature importance is ranked in terms of logarithmic mean absolute SHAP values. SHAP values relative to the average number of previous COVID-19 cases as a predictor for the current number of COVID-19 cases were computed but have been omitted in Figs 6 and 7 due to their obvious relevance in order to focus on the other variables. All meteorological and air quality factors score at similar levels of importance, showing that there is no dominant predictor. UV radiation is the meteorological factor with the greatest SHAP value, confirming the results of the statistical analysis where UV radiation was the most highly correlated factor with COVID-19 cases. Socioeconomic, environmental, and global health indicator variables all show minor impacts other than population density, and the annual carbon dioxide emissions (a time-invariant proxy for the country’s overall air quality). Intervention and health system policies, described by the different OxCGRT indices, all score similar to or slightly lower than meteorological factors. Although not included in Fig 6, the average number of previous COVID-19 cases results having the greatest impact (i.e. highest SHAP value) in line with results reported in the current literature on COVID-19 and other coronaviruses [80].

Fig 7 shows the intensity and polarity of specific SHAP variable values for each data point (a dot in the plot) with reference to the dependent variable (daily COVID-19 cases). The red-to-blue color scale indicates magnitude (high/red vs. low/blue). Position on the horizontal axis signals polarity (negative vs. positive).

The resulting analysis suggests that higher UV radiation is significantly correlated with lower occurrence of COVID-19 cases (the dependent variable), while the other meteorological factors show a much weaker contribution. Temperature shows a weakly positive correlation, but the results are not consistent. Absolute humidity also displays a weakly positive correlation, despite the expected impact according to earlier studies. Rainfall appears to be negatively correlated with the dependent variable, but there are too few observations for high rainfall values in the dataset to properly confirm this result. All the other meteorological factors do not exhibit a distinct correlation directionality or significant impact.

Among the intervention policies, both the OxCGRT stringency and containment & health indices display a weakly negative correlation suggesting that more stringent prevention measures have been beneficial in mitigating the spread of COVID-19, at least for a significant number of countries. Policies on school closures, stay-at-home requirements, and testing reveal a somewhat lower weakly negative correlation.

In order to provide a more detailed analysis, we have also compared feature importance analysis results of locations in the northern and southern hemispheres. As the SHAP values in S3 Fig show, the results for each hemisphere are in line with those of the global analysis (Fig 6). Meteorological conditions are still the more crucial factors when compared to intervention policies, with UV still among the most prominent features for both datasets. As shown in S4 Fig, the intensity and polarity of SHAP values for the explanatory variables in separate hemisphere are also in line with those for the entire globe (Fig 7), although for some variables in the southern hemisphere polarity this similarity is not as explicit as for the northern hemisphere. For example, while UV still shows a negative association in both hemispheres, the distribution of SHAP score for the southern hemisphere is less marked than in the northern hemisphere. By contrast, rainfall displays a clear negative correlation in the southern hemisphere that is not clearly visible in the northern hemisphere.

For completeness, we include the feature importance results for the Lasso, Elastic Net, and random forest models at a global scale (S5 Fig). All point to UV as one of the most important parameters. For Lasso and Elastic Net we report regression coefficients. For the random forest tree, we use the Gini importance score.

### Econometric analysis

The econometric analysis was carried out using a panel data fixed-effects model. Confirmed daily cases of COVID-19 in log-scale were regressed against climate and air-quality factors, with reference to cross-sectional and time fixed effects. We could not add in the regression the moving averages of the dependent variable as we did for the machine learning analysis because this inclusion would violate the assumptions underlying the fixed effects estimator. If the independent variable is correlated with the error term in a regression model (endogeneity), then the estimate of the regression coefficient would be inconsistent. Moreover, adding one or more autocorrelated terms to the regression would remove most of the model variance, making the effects of the other independent variables less significant (leading to smaller *β*_*n*_ and larger standard errors).

Note that the nominal magnitude of the regression coefficient of every single explanatory variable is likely to be biased due to the undetermined confounding effects. For this reason, we mainly focus our discussion of the results on the significance and polarity of the coefficients.

Before proceeding with the econometric analysis, we test our data for stationarity, since non-stationary data may lead to spurious regression results thus falsely indicating the existence of a relationship between two variables [81]. For each time series variable considered in the econometric study, we run the Fisher-type unit-root test based on augmented Dickey-Fuller tests at 0 and 1 lag. Table 3 provides a summary of the results for the variables with moving averages at 7 days. We omit the test results for other window sizes for which we obtain the same outcome. The four tests all strongly reject the null hypothesis that all the panels contain unit roots for each variable under consideration and we can therefore proceed with the analysis.

**Table 3:** Results of the Fisher-type unit-root test analysis based on augmented Dickey-Fuller tests at 0 and 1 lag for each variable considered in the econometric study.

	I(0) 0 Lags				I(1) 1 Lag			
	Inverse chi-squared	Inverse normal	Inverse logit t	Modified inv. chi-squared	Inverse chi-squared	Inverse normal	Inverse logit t	Modified inv. chi-squared
	P	Z	L*	Pm	P	Z	L*	Pm
Daily cases (log)	9101.3	-74.1	-106.8	171.2	5631.1	-53.5	-67.5	101.6
Temperature_7	1868.7	-14.4	-14.2	16.5	3157.1	-33.9	-35.7	47.0
Absolute Humidity_7	1912.3	-15.2	-14.8	17.4	3546.0	-36.7	-40.4	55.6
Pressure_7	3112.6	-29.9	-31.9	43.1	8835.9	-69.5	-106.1	172.4
Wind speed_7	4163.5	-40.5	-46.5	65.6	7312.3	-62.4	-87.8	138.8
Rainfall_7	4974.1	-43.9	-54.1	82.9	6797.6	-57.4	-80.1	127.4
Short-wave irradiation_7	2599.7	-26.5	-26.9	32.2	3156.0	-34.0	-36.0	47.0
PM2P5_7	3763.2	-36.7	-40.9	57.1	8004.9	-65.7	-96.0	154.0
PM10_7	3765.8	-36.2	-40.9	57.1	7778.1	-63.4	-92.9	149.0
UV_7	2516.5	-25.4	-25.7	30.4	3184.9	-33.6	-36.2	47.7
High_stringency_7	3333.0	-40.3	-50.0	73.7	2805.7	-36.5	-43.6	62.9
High_containment_7	4123.7	-45.5	-59.62	90.4	3302.7	-39.9	-50.2	74.4

For each test, we report inverse chi-squared, inverse normal, inverse logit t, and modified

The statistical significance of the regression coefficients is computed by clustering the regression standard error at the country/admin level, to account for error correlation within the geographical areas where our unit of observation was collected. For each location, we select days with a minimum of 10 confirmed COVID-19 cases, and we limit our analysis to the locations with at least 90 data records (3 months’ worth of data), in line with the other analyses. The results are reported in Table 4 for all the T-day moving averages and time-variant regressors (see Methodology section). The regression model has a R^2^ value of 0.73 over 65,369 observations, which shows that the independent and dependent variables are significantly correlated. UV radiation shows strong negative correlation with COVID-19 spread, while temperature has a positive association, in line with the statistical correlation and feature importance analyses discussed in the previous two sections. Both results are statistically significant (P < 0.01). For other climatic factors, the econometric analysis is congruent with the feature importance analysis, but either the coefficients are not statistically significant, or the magnitude of their regression coefficients is comparable to the standard error (e.g., PM2.5 and PM10). Rainfall seems to have significance only at shorter moving averages (5 days). For the remaining meteorological factors, different moving averages (5, 7, 10, 12, and 14 days), which relate climatic variables to incubation periods of diverse duration, do not seem to influence the overall result of the econometric analysis.

**Table 4:** Panel data fixed-effects model.

Dependent variable:	(1)	(2)	(3)	(4)	(5)
Daily cases (log)	T = 5	T = 7	T = 10	T = 12	T = 14
Days_from_start	0.006***	0.006***	0.006***	0.006***	0.006***
	(0.00)	(0.00)	(0.00)	(0.00)	(0.00)
Temperature_T	0.022***	0.025***	0.028***	0.030***	0.032***
	(0.01)	(0.01)	(0.01)	(0.01)	(0.01)
Absolute Humidity_T	-0.011	-0.012	-0.013	-0.015	-0.016
	(0.02)	(0.02)	(0.02)	(0.02)	(0.02)
Pressure_T	-0.004	-0.004	-0.005	-0.005	-0.005
	(0.00)	(0.00)	(0.01)	(0.01)	(0.01)
Windspeed_T	-0.031	-0.036	-0.040	-0.043	-0.046
	(0.02)	(0.02)	(0.03)	(0.03)	(0.04)
Rainfall_T	-0.010**	-0.009	-0.008	-0.007	-0.006
	(0.00)	(0.01)	(0.01)	(0.01)	(0.01)
Shortwave Irradiation_T	-0.000	-0.000	-0.000	-0.000	-0.000
	(0.00)	(0.00)	(0.00)	(0.00)	(0.00)
PM 2.5_T	-0.014***	-0.016***	-0.018***	-0.019***	-0.020***
	(0.00)	(0.00)	(0.00)	(0.00)	(0.01)
PM 10_T	0.003***	0.003***	0.004***	0.004***	0.004***
	(0.00)	(0.00)	(0.00)	(0.00)	(0.00)
UV_T	-0.492***	-0.543***	-0.607***	-0.634***	-0.657***
	(0.09)	(0.11)	(0.13)	(0.14)	(0.15)
Constant	7.283**	7.648**	8.128**	8.473*	8.860**
	(3.12)	(3.51)	(4.06)	(4.37)	(4.69)
**Observations**	65,369	65,369	65,369	65,369	65,369
***R*** ^**2**^	0.734	0.735	0.736	0.736	0.737
**Adjusted *R*** ^**2**^	0.733	0.734	0.735	0.735	0.736

T-days moving average. Standard errors in parentheses are clustered at location (country/region) level,

* p < 0.10,

** p < 0.05,

*** p < 0.01.

Testing for the added effect of intervention policies requires a more in-depth analysis. The enactment of restrictions and the pandemic peak tend to vary from country to country due to the diversity, severity, and enforcement ability of the containment policies implemented. For this reason, we limit our analysis to the OxCGRT stringency and containment & health indices which allow us to capture the general level of restriction without focusing on the intervention policies of each government. Moreover, most countries have applied distinct levels of restrictions at a much deeper level of granularity than what the available data would allow us to test for (city, province, or regional level). Therefore, we only present an analysis of the north American region including all the US states and Canadian territories, for which OxCGRT provides the highest level of detail.

Table 5 provides the results of panel data analysis on the impact of stringency and containment levels for COVID-19 and climatic factors. To evaluate the impact of the stringency factor, we created the two variables high_stringency and high_containment that take values equal to 1 when stringency and the containment & health are above 60% (median value) and 0 otherwise. We separately test the lagged effect of these two factors at 7 and 14 days as expressed by the numerical suffix associated with the two variables in Table 5. We find that high levels of closure-type restrictions show significant effects on limiting COVID-19 spread only after about two weeks from their introduction (P < 0.01). Conversely, the containment & health index presents a strong negative regression coefficient at both 7 and 14 days from introduction (P < 0.01). This may be related to the added efficacy of combining different health and prevention policies (public info campaigns, PCR testing, contact tracing, and facial coverings) to enable a faster control on the containment of viral transmission. The R^2^ coefficient of 0.73 over 19,289 observations indicates a high level of correlation for this regression model. The robustness of our results is corroborated by the fact that both polarity and magnitude of the regression coefficients for the climate variables are still in line with statistical correlation and feature importance results, despite having developed the regression model with a smaller pool of data and additional factors.

**Table 5:** Panel data fixed-effects model—testing the effect of restrictions.

Dependent variable:	(1)	(2)	(3)	(4)
Daily cases (log)				
days_from_start	0.006***	0.006***	0.006***	0.006***
	(0.00)	(0.00)	(0.00)	(0.00)
Temperature_7	0.024***	0.020***	0.022***	0.017***
	(0.01)	(0.01)	(0.01)	(0.01)
Absolute Humidity_7	-0.028	-0.023	-0.028	-0.021
	(0.02)	(0.02)	(0.02)	(0.02)
Pressure_7	-0.024***	-0.025***	-0.025***	-0.023***
	(0.01)	(0.00)	(0.00)	(0.00)
Wind speed_7	0.030	0.030	0.029	0.031
	(0.02)	(0.02)	(0.02)	(0.02)
Rainfall_7	-0.013	-0.010	-0.013	-0.013
	(0.01)	(0.01)	(0.01)	(0.01)
Short-wave irradiation_7	-0.000	-0.000	-0.000	-0.000
	(0.00)	(0.00)	(0.00)	(0.00)
PM2P5_7	0.002	0.004	0.001	0.001
	(0.01)	(0.01)	(0.01)	(0.01)
PM10_7	-0.003	-0.002	-0.004	-0.004
	(0.01)	(0.01)	(0.01)	(0.01)
UV_7	-0.376**	-0.374**	-0.372**	-0.362**
	(0.17)	(0.17)	(0.17)	(0.17)
High_stringency_7	-0.087			
	(0.07)			
High_stringency_14		-0.273***		
		(0.07)		
High_containment_7			-0.176***	
			(0.07)	
High_containment_14				-0.388***
				(0.07)
Constant	28.414***	28.456***	28.351***	26.469***
	(4.28)	(4.23)	(4.36)	(4.20)
**Observations**	19,289	19,289	19,289	19,289
***R*** ^**2**^	0.733	0.736	0.734	0.741
**Adjusted *R*** ^**2**^	0.732	0.736	0.733	0.740

Results only for the Canadian territories and the United States. Standard errors in parentheses are clustered at location (country/region) level,

* p < 0.10,

** p < 0.05,

*** p < 0.01.

Results in Tables 4 and 5 are presented in tabular format by listing the values of the intercept (constant) and the *β* coefficients with their standard error for each regressor under the different hypotheses of duration relative to various moving average window sizes (T).

## Discussion

Table 6 summarizes the results about the impact of climate factors on COVID-19 transmission from the three types of analysis carried out in this study (statistical, machine learning, and econometric analyses). Scores are color-coded to indicate the positive (red), negative (blue), or undetermined (black) polarity of variables correlated with COVID-19 transmission. Relative ranking per analytic method is indicated by integers enclosed within parentheses. For the statistical analysis, we report the median magnitude of Spearman’s coefficient, and we rank the results based on their absolute value. For the machine learning analysis, we relay feature importance and its ranking as the mean absolute SHAP value. For the econometric analysis, we look at the significance of each estimated regression coefficient and we rank them based on their magnitude normalized with reference to their standard error. As discussed in the introduction, we include the statistical analysis as a baseline, leverage the machine learning analysis for short-term prediction, and use the econometric results as an indication of long-term trends.

**Table 6:** Coefficients and relative rank describing the impact of climate factors on COVID-19 transmission across the three types of analysis carried out in this study.

	Statistical analysis (Spearman’s coefficient)	Machine learning analysis (SHAP values)	Econometric analysis (Panel data fixed effects model)
Temperature	$-0.42\left(3\right)$	$14.7\left(2\right)$	$***\text{p}>0.10\left(5\right)$
Absolute Humidity	$-0.39\left(4\right)$	$11.6\left(4\right)$	$\text{p}>0.10\left(7\right)$
Pressure	$0.19\left(8\right)$	14.5 (3)	$***\text{p}<0.001\left(3\right)$
Wind speed	$0.21\left(6\right)$	10.4 (8)	$\text{p}>0.10\left(6\right)$
Rainfall	$-0.21\left(7\right)$	$10.3\left(9\right)$	$\text{p}<0.10\left(8\right)$
Short-wave irradiation	$-0.55\left(2\right)$	$10.9\left(5\right)$	p > 0.10 (11)
PM2.5	$0.24\left(5\right)$	10.5 (7)	$\text{p}>0.10\left(9\right)$
PM10	$0.17\left(9\right)$	$10.8\left(6\right)$	$\text{p}>0.10\left(10\right)$
UV	$-0.56\left(1\right)$	$18.1\left(1\right)$	$**\text{p}<0.001\left(4\right)$
Stringency		$8,8\left(10\right)$	$***\text{p}<0.001\left(2\right)$
Containment		$7.9\left(11\right)$	$***\text{p}<0.001\left(1\right)$

Red fonts indicate negative correlations, blue positive correlations, and black undetermined polarity. The integers enclosed in parentheses describe relative rank (1 = highest, 11 = lowest). Stringency and containment results are not available in the statistical analysis.

SHAP values for meteorological and environmental factors other than UV tend to cluster very closely (see Fig 6), so their relative ranking points to relatively mild impact differences. Also, only pressure, temperature, UV, stringency, and containment factors show sufficient statistical significance (p < 0.10) in the econometric results. Results for rainfall, absolute humidity, wind speed, short-wave irradiation, PM2.5, and PM10 where p > 0.10 can only be seen as weak indicators.

UV emerges as the most impactful meteorological factor in COVID-19 transmission across all methods. More specifically, UV is negatively correlated with COVID-19 spread. This result is corroborated by overwhelming evidence that UV light can effectively kill SARS-CoV-2 and other coronaviruses [82–84].

Temperature is positively correlated with COVID-19 transmission in both the machine learning and econometric analyses. As discussed in the introduction, there is contrasting evidence about the role of temperature in the spread of COVID-19. The inverse correlation of UV and temperature with COVID-19 spread emerging in our analysis suggests that the inhibiting factor in those studies where the temperature is negatively correlated with COVID-19 spread may not temperature per se, but rather the high UV that is often found in hotter climates. This is a hypothesis that requires further inspection.

Absolute humidity is positively correlated with COVID-19 spread in the machine learning analysis and negatively correlated in the econometric analysis, though with insufficient statistical significance. This is an interesting contrast as there is conflicting evidence in the literature about the role of absolute humidity in the spread of COVID-19, as discussed in the introduction (S1 Table). Perhaps, the two analyses point to a different role that absolute humidity may play in the short and long term with reference to COVID-19 spread. This too is a hypothesis that requires further inspection.

PM10, stringency, and containment all show the same polarity in the machine learning and econometric analysis, though only for containment and stringency the econometric analysis yields statistical relevance (p < 0.001).

Discrepancies between results in the statistical analysis and the machine learning and econometric analyses emphasize the difficulty in statistical analysis to deal with non-stationary processes and account for long terms trends. Perhaps, the most interesting discrepancies are those between machine learning and econometric analysis. As discussed in the introduction, machine learning is better equipped to take advantage of structural heterogeneity in training data to make short-term predictions, whereas econometric methods are better at capturing long-term trends [73]. Therefore, diverging results across the two methods may be indicative of short-term vs. long-term impacts.

## Conclusions

Overall, disease susceptibility is the main factor driving the pandemic growth. Compliance with lockdown and restrictions policies and regulations and increased testing are the most effective strategies for disease control and COVID-19 spread prevention. For example, various studies have reported that interventions such as restrictions on mass gatherings, school closures, and social distancing measures are strongly associated with a decrease in the COVID-19 transmission growth rate [33, 38, 39, 64, 85–87]. The correlation of COVID-19 transmission with climate factors provides a valuable complementary diagnostic that sheds light on the seasonal characterization of the pandemic and helps refine measures to contain and prevent the spread of COVID-19. More specifically, weather forecasts could help predict new cycles of the pandemic and future outbreaks and thus contribute to the definition of ad-hoc measures that limit the economic impact of complete lockdowns. This study also extends the reach of earlier studies (S1 Table) on the relationship between COVID-19 transmission and climate factors by assessing how climate helps modeling COVID-19 through systematic validation using statistical, feature importance, and econometric analyses. Such validation is crucial in proving which are the contributing factors and their relative magnitude and direction of change.

## Supporting information

S1 File (DOCX)

S1 Fig Statistical distribution of the share of population emerging from the sampling approach of meteorological conditions adopted in this study. On the left is a frequency histogram of the population share covered by the sampling approach for each county. For most of the countries the share is above 75% and for only few of them is less than 20%. On the right side of the figure is a scatter plot of the share for each location considered in the study as a function of the overall population size.
(TIF)

S2 Fig Summary diagram of the methodological approach. (TIF)

S3 Fig Feature importance summary plot for the north and south hemispheres. Mean absolute SHAP value (in log scale) of each variable showing the average impact on the model output magnitude for the locations in the north (left) and south (left) hemispheres.
(TIF)

S4 Fig Feature impact scatters plot for the north and south hemispheres. SHAP value of each variable for all the single observations as a function of their relative value for the locations in the north (left) and south (left) hemispheres. The color transition on the vertical axis indicates value strength (red/high to blue/low).
(TIF)

S5 Fig Feature importance analysis based on Lasso, Elastic Net, and Random Forest algorithms. For Lasso and Elastic Net we report their regression coefficients (red for negative values and blue for positive). For the random forest tree, we use the Gini importance score.
(TIF)

S1 Table Summary of peer-reviewed literature on research studies on the interrelationship between COVID-19 and environmental/climatic factors. (DOCX)

S2 Table Description of the variables used for the study. (DOCX)

S3 Table Descriptive statistics. (DOCX)
