# Supplementary material for: Does climate help modeling COVID-19 risk and to what extent?
Source: PLoS One. 2022 Sep 7;17(9):e0273078. doi: 10.1371/journal.pone.0273078 (PMC9451080; doi:10.1371/journal.pone.0273078)
Supplement: S1 File — (DOCX) [file pone.0273078.s001.docx]

# Supporting information

**1. Data available at regional-, county-, or province-level for the following countries ^a^:** Czechia, Denmark, Estonia, France, Ireland, Italy, Japan, Latvia, Lithuania, Netherlands, Panama, Poland, South Africa, South Korea, Spain, Sweden, Ukraine.

**2. Data available at state-level for the following countries ^a,b^:** Australia, Austria, Brazil, Canada, China, Germany, India, Russia, Switzerland, United States, United Kingdom.

**3. Data available at country-level for the following countries:** Afghanistan, Albania, Algeria, Andorra, Angola, Antigua and Barbuda, Argentina, Armenia, Australia, Austria, Azerbaijan, Bahamas, Bahrain, Bangladesh, Barbados, Belarus, Belgium, Belize, Benin, Bhutan, Bolivia, Bosnia and Herzegovina, Botswana, Brazil, Brunei, Bulgaria, Burkina Faso, Burma, Burundi, Cabo Verde, Cambodia, Cameroon, Canada, Central African Republic, Chad, Chile, China, Colombia, Comoros, Congo (Brazzaville), Congo (Kinshasa), Costa Rica, "Cote d’Ivoire", Croatia, Cuba, Cyprus, Czechia, Denmark, Djibouti, Dominican Republic, Ecuador, Egypt, El Salvador, Equatorial Guinea, Eritrea, Estonia, Eswatini, Ethiopia, Finland, France, Gabon, Gambia, Georgia, Germany, Ghana, Greece, Guam, Guatemala, Guinea, Guinea-Bissau, Guyana, Haiti, Honduras, Hungary, Iceland, India, Indonesia, Iran, Iraq, Ireland, Israel, Italy, Jamaica, Japan, Jordan, Kazakhstan, Kenya, Korea, South, Kosovo, Kuwait, Kyrgyzstan, Latvia, Lebanon, Lesotho, Liberia, Libya, Liechtenstein, Lithuania, Luxembourg, Madagascar, Malawi, Malaysia, Maldives, Mali, Malta, Mauritania, Mauritius, Mexico, Moldova, Monaco, Mongolia, Montenegro, Morocco, Mozambique, Namibia, Nepal, Netherlands, New Zealand, Nicaragua, Niger, Nigeria, North Macedonia, Norway, Oman, Pakistan, Panama, Papua New Guinea, Paraguay, Peru, Philippines, Poland, Portugal, Puerto Rico, Qatar, Romania, Russia, Rwanda, San Marino, Sao Tome and Principe, Saudi Arabia, Senegal, Serbia, Seychelles, Sierra Leone, Singapore, Slovakia, Slovenia, Somalia, South Africa, South Sudan, Spain, Sri Lanka, Sudan, Suriname, Sweden, Switzerland, Syria, Taiwan, Tajikistan, Tanzania, Thailand, Togo, Trinidad and Tobago, Tunisia, Turkey, United States, Uganda, Ukraine, United Arab Emirates, United Kingdom, United States Virgin Islands, Uruguay, Uzbekistan, Venezuela, Vietnam, West Bank and Gaza, Yemen, Zambia, Zimbabwe.

^a^ We use “admin-level” as a general categorical description of this type of location to distinguish it from group #3 in the main text of the manuscript.

^b^ We list locations for which data is available at a lower administrative level but still higher than regional-, county-, or province-level. While for most of the listed countries the use of “state” for their political sub-units is correct, for Canada we refer to its territories, Switzerland to its cantons, and for the United Kingdom to its countries.
