# Supplementary figures and images for: Does climate help modeling COVID-19 risk and to what extent?
Source: PLoS One. 2022 Sep 7;17(9):e0273078. doi: 10.1371/journal.pone.0273078 (PMC9451080; doi:10.1371/journal.pone.0273078)

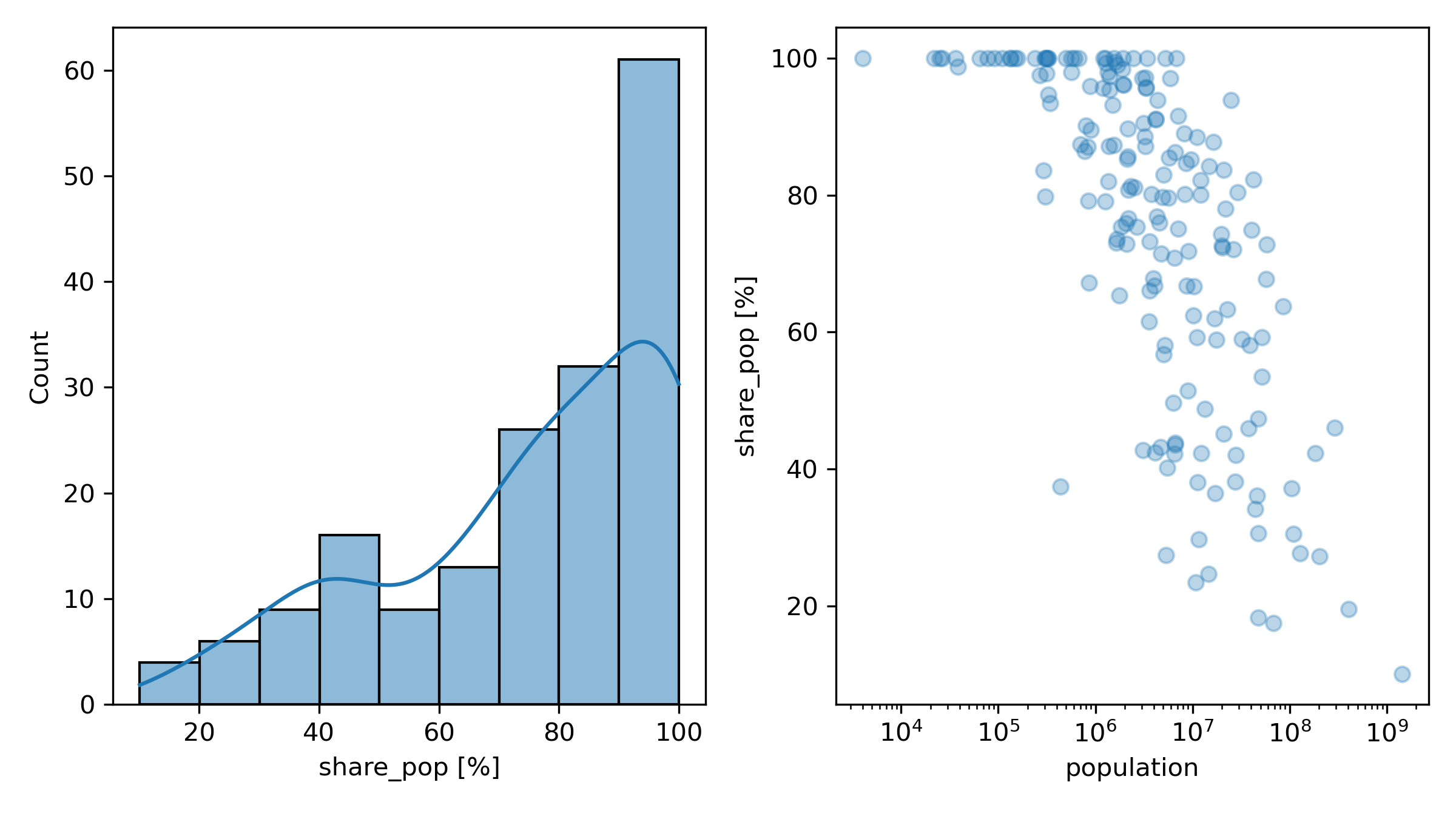

Supplement: S1 Fig — On the left is a frequency histogram of the population share covered by the sampling approach for each county. For most of the countries the share is above 75% and for only few of them is less than 20%. On the right side of the figure is a scatter plot of the share for each location considered in the study as a function of the overall population size. (TIF) [file pone.0273078.s002.tif]

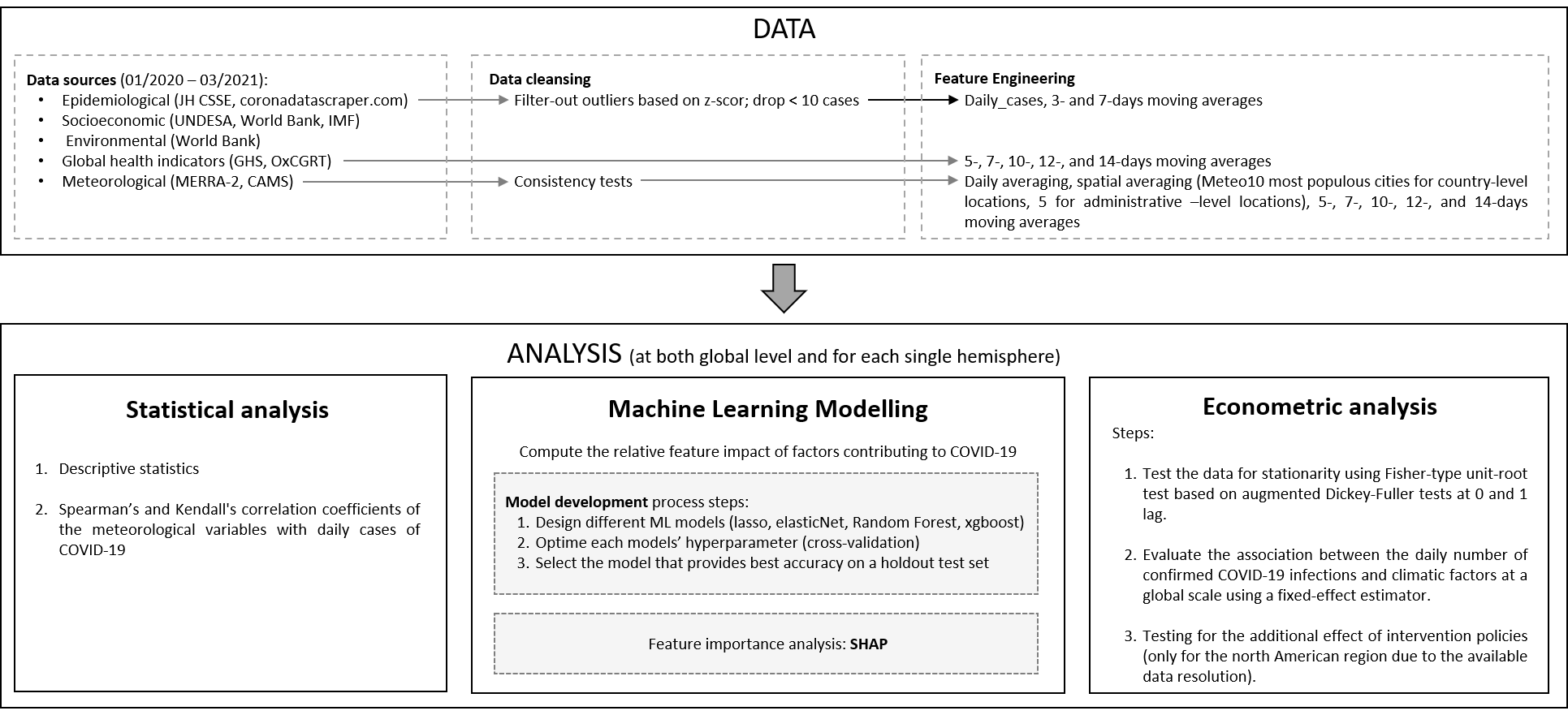

Supplement: S2 Fig — (TIF) [file pone.0273078.s003.tif]

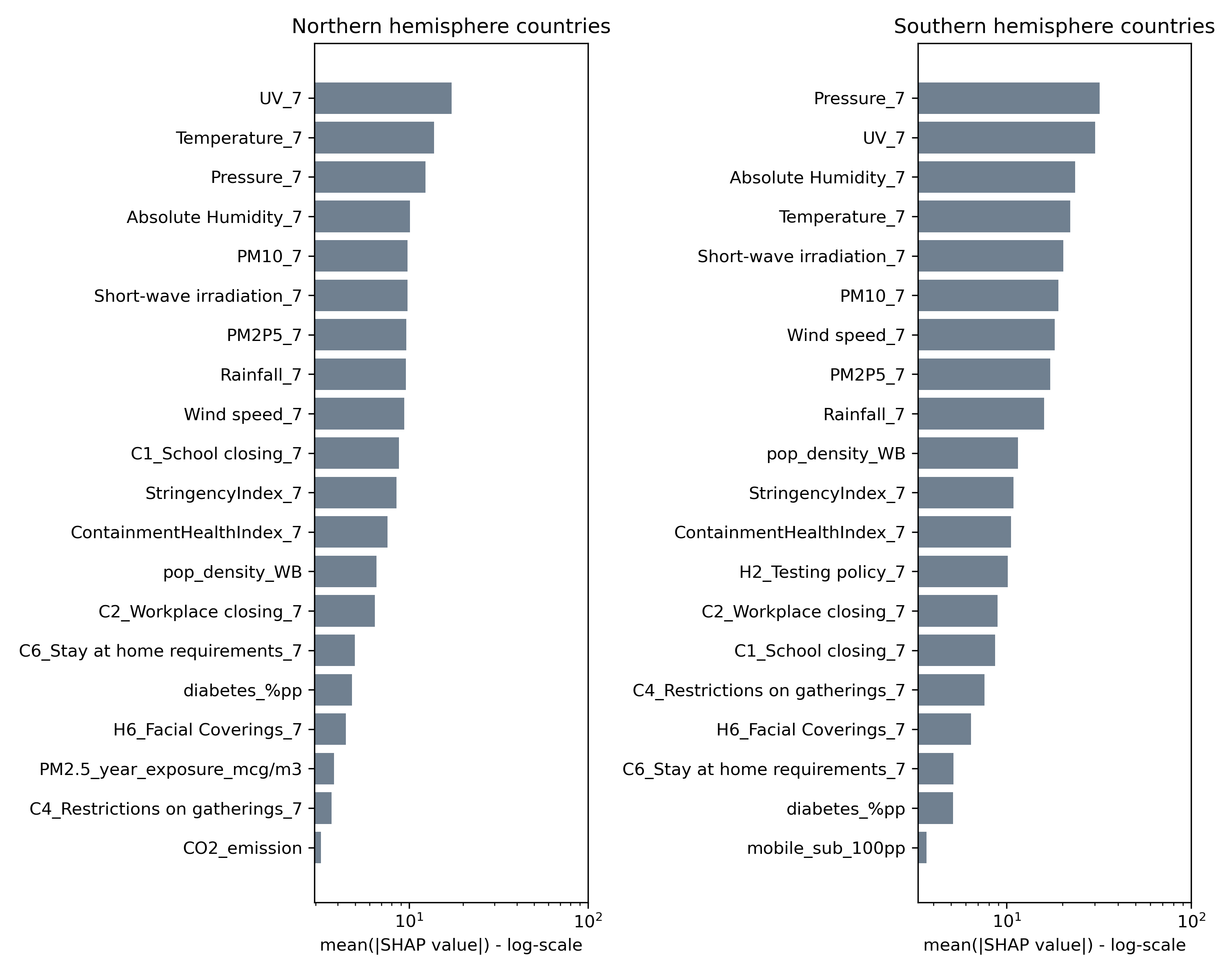

Supplement: S3 Fig — Mean absolute SHAP value (in log scale) of each variable showing the average impact on the model output magnitude for the locations in the north (left) and south (left) hemispheres. (TIF) [file pone.0273078.s004.tif]

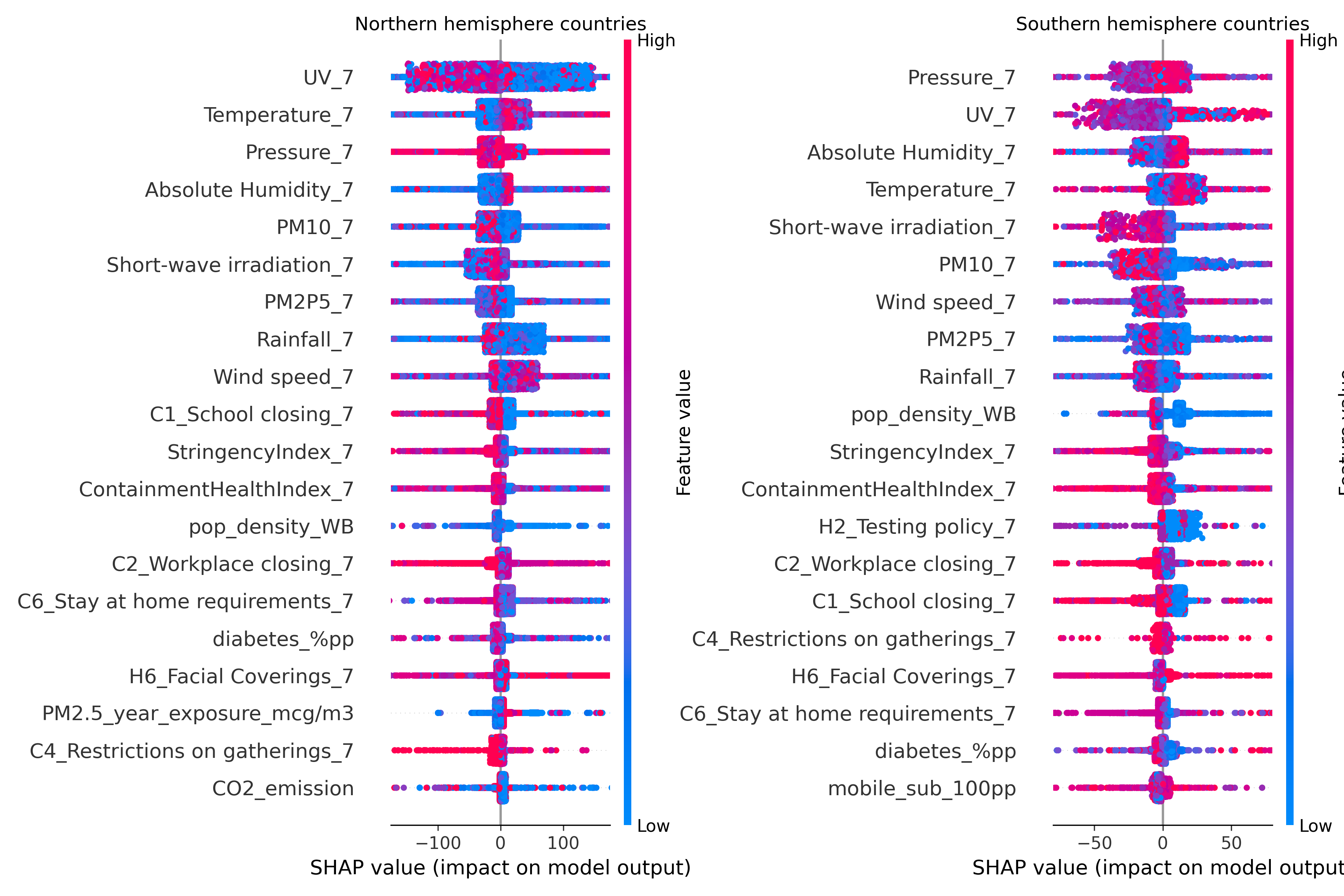

Supplement: S4 Fig — SHAP value of each variable for all the single observations as a function of their relative value for the locations in the north (left) and south (left) hemispheres. The color transition on the vertical axis indicates value strength (red/high to blue/low). (TIF) [file pone.0273078.s005.tif]

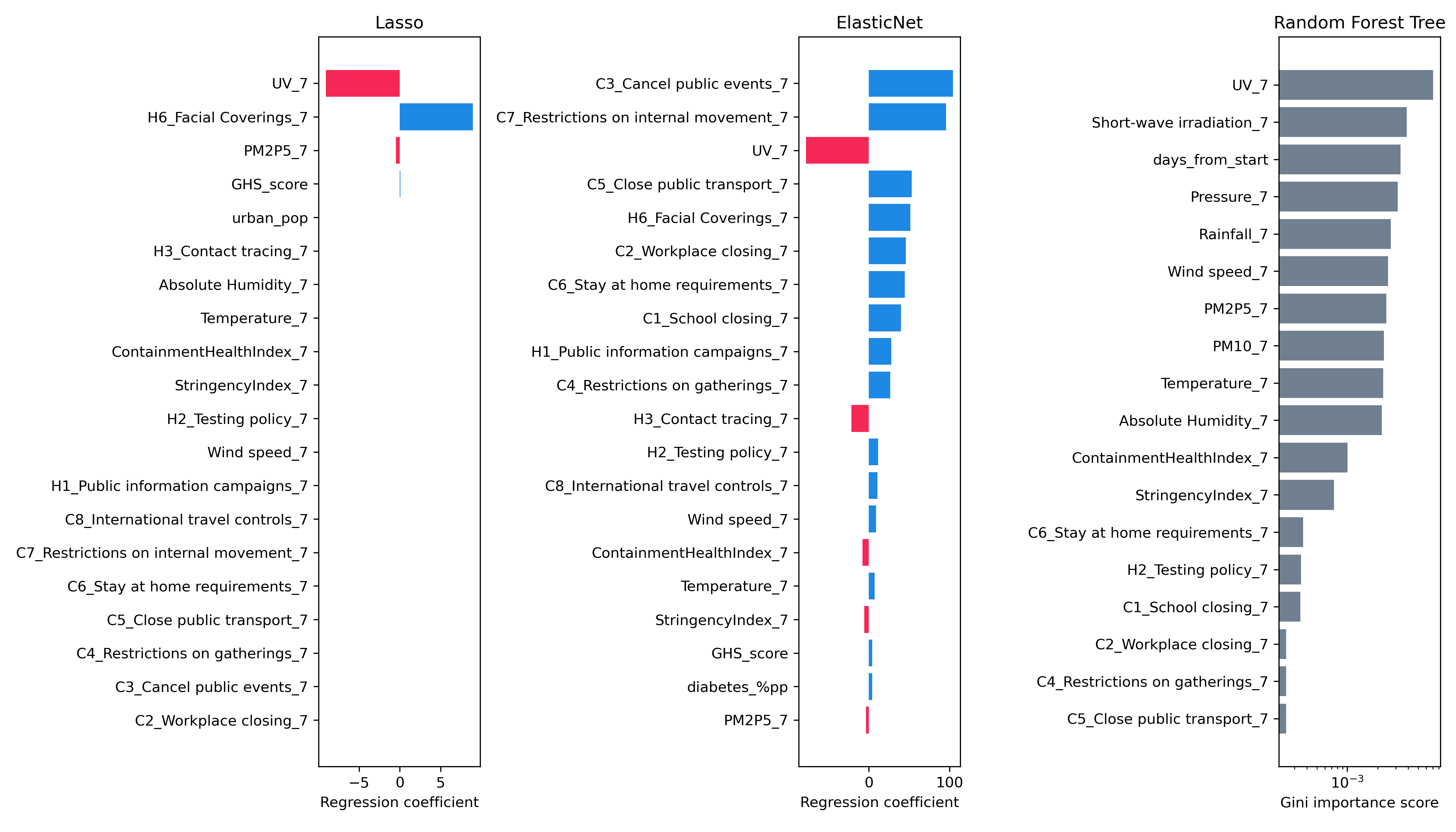

Supplement: S5 Fig — For Lasso and Elastic Net we report their regression coefficients (red for negative values and blue for positive). For the random forest tree, we use the Gini importance score. (TIF) [file pone.0273078.s006.tif]
