# Supplementary material for: Does climate help modeling COVID-19 risk and to what extent?
Source: PLoS One. 2022 Sep 7;17(9):e0273078. doi: 10.1371/journal.pone.0273078 (PMC9451080; doi:10.1371/journal.pone.0273078)
Supplement: S1 Table — (DOCX) [file pone.0273078.s007.docx]

**S1 Table. Summary of peer-reviewed literature on research studies on the interrelationship between COVID-19 and environmental/climatic factors.**

| **REF** | **Location setting** | **Duration** | **Methodology** | **PM2.5** | **PM10** | **Air quality**  **(pollution)** | **Humidity** | **Temp.** | **Rain** | **Wind speed** | **Solar / UV** |
| --- | --- | --- | --- | --- | --- | --- | --- | --- | --- | --- | --- |
| [10] | Cities of Wuhan, Xiaogan, and Huanggang, China | 1 month | Multivariate Poisson regression | positive | negative | mixed | positive | negative |  | - |  |
| [11] | 30 Chinese provinces | 1 month | Generalized Additive Model (GAM) |  |  |  | mixed /  negative | mixed / negative |  |  |  |
| [12] | 100 Chinese cities | 1 month | Regression |  |  |  | negative | negative |  |  |  |
| [13] | 30 provincial capital cities of China | 2 months | Non-linear regression and generalized linear models with negative binomial distribution |  |  |  | negative | negative |  |  |  |
| [14] | New York City | 1 month | Kendall and Spearman rank correlation |  |  | negative | - | positive | - | - |  |
| [15] | China prefectures | 2 months | Two-way fixed effect model |  |  |  | negative | negative |  |  |  |
| [16] | Beijing | 3 months | Multiple stepwise linear regression |  |  |  | negative | negative |  | - |  |
| [17] | Italian cities (global) | 3 months | Maximum Entropy-based Ecological Niche Model |  |  | positive |  | negative | negative |  |  |
| [18] | Italy (20 regions and 107 provinces) | - | Multivariate OLS regression | positive | positive | positive | positive | negative |  |  |  |
| [19] | Spain (province level) | 5 months | Linear regression model |  |  |  |  | negative | - |  | - ^a^ |
| [20] | 31 states and capital of Mexico | 1 month | Spearman's non-parametric test |  |  |  |  | negative | positive |  |  |
| [21] | 27 state capitals of Brazil | 9 months | Two-way fixed-effect models |  |  |  | Mixed / negative | Mixed / negative | - | negative |  |
| [22] | Latin America and the Caribbean (LAC) region | 2 months | Spearman rank correlation tests | mixed | mixed | positive | positive | negative | mixed | negative |  |
| [23] | Global | 3 months | Panel data model |  |  |  |  | negative | positive |  |  |
| [24] | Global | 3 months | Distributed lag non-linear model |  |  |  | negative | negative |  | negative |  |
| [25] | Global | 1 month | Exponential fit |  |  |  |  | negative |  |  |  |
| [26] | Global | 1 month | Global statistical analysis, Bivariate time-series analysis with Scale-dependent correlations, MSDC analysis |  |  |  | negative | negative |  |  |  |
| [27] | 359 cities – global | 6 months | OLS model |  |  | positive | - |  |  |  | negative |
| [28] | 122 cities in China | 2 months | Generalized additive model (GAM) |  |  |  |  | positive |  |  |  |
| [29] | 5 Brazilian cities | 1 month | Multivariate statistical analysis, linear regression |  |  |  | mixed / positive | mixed / positive | mixed / positive |  |  |
| [30] | Oslo, Norway | 2 months | Non-parametric correlation test |  |  |  |  | positive | negative | - |  |
| [31] | Indonesia | 1 month | Spearman-rank correlation test |  |  |  | - | positive | - |  |  |
| [32] | New South Wales, Australia | 3 months | Generalized additive model (GAM) |  |  |  | negative | - | - |  |  |
| [33] | Global | 3 months | Weighted random-effects regression |  |  |  | negative | - |  |  |  |
| [34] | 134 countries | 15 months | Panel linear regression analysis |  |  |  | negative | - | - | - |  |
| [40] | Italy | 2 months | Correlation (bivariate and partial) and regression analyses |  | positive | positive |  |  |  | negative |  |
| [41] | Italy | 2 months | Correlation analysis, regression analysis |  | positive | positive |  |  |  | negative |  |
| [42] | 81 provinces in Turkey | 1 month | Regression analysis |  |  | - | - | - |  | positive | - ^b^ |
| [43] | State of Rio de Janeiro, Brazil (6 cities) | 2 months | Spearman-rank correlation test |  |  |  |  | negative |  | negative | negative |
| [44] | Australia, state of Victoria | 9 months | Statistical Analysis and linear regression model |  |  |  |  | - | - |  | negative |
| [45] | 12 countries | 11 months | Automated machine learning (ML) |  |  |  |  |  |  |  | negative |
| [46] | 67 countries | 12 months | Seasonality analysis |  |  |  | - | - |  |  | negative |

Only statistically significant (p < 0.01) associations are considered: either positive or negative correlation. Mixed is reported when the results are ambiguous across the factors under investigation or within the considered locations. “-“ is used for factors considered in the study that are not statistically significant. ^a^ Daily sun hours, ^b^ number of sunny days,
