# Supplementary material for: Does climate help modeling COVID-19 risk and to what extent?
Source: PLoS One. 2022 Sep 7;17(9):e0273078. doi: 10.1371/journal.pone.0273078 (PMC9451080; doi:10.1371/journal.pone.0273078)
Supplement: S2 Table — (DOCX) [file pone.0273078.s008.docx]

**S2 Table. Description of the variables used for the study.**

| **Variable name** | **Data source** | **Type** | **Description** |
| --- | --- | --- | --- |
| daily_cases | Johns Hopkins and the Corona Data Scraper | Time series | Daily cases of COVID-19 |
| days_from_start | Calculated | Time series | Number of days from first case |
| Temperature | MERRA-2 | Time series | In °C. Daily average at 2m above ground |
| Absolute humidity | MERRA-2 | Time series | In g/m^3^. Calculated from temperature and relative humidity |
| Pressure | MERRA-2 | Time series | In hPa. At ground level (station-level) |
| Wind speed | MERRA-2 | Time series | In m/s. At 10m above ground |
| Rainfall | MERRA-2 | Time series | In mm. |
| Short-wave irradiation | MERRA-2 | Time series | In Wh/m^2^. Surface downward short-wave irradiation (broadband) |
| PM2P5 | CAMS | Time series | In 𝜇g/m^3^ |
| PM10 | CAMS | Time series | In 𝜇g/m^3^ |
| UV | CAMS | Time series | In MJ/m^2^ |
| C1_School closing* | OxCGRT | Ordinal time series | Record closings of schools and universities |
| C2_Workplace closing* | OxCGRT | Ordinal time series | Record closings of workplaces |
| C3_Cancel public events* | OxCGRT | Ordinal time series | Record cancelling public events |
| C4_Restrictions on gatherings* | OxCGRT | Ordinal time series | Record limits on gatherings |
| C5_Close public transport* | OxCGRT | Ordinal time series | Record closing of public transport |
| C6_Stay at home requirements* | OxCGRT | Ordinal time series | Record orders to "shelter-in-place" and otherwise confine to the home |
| C7_Restrictions on internal movement* | OxCGRT | Ordinal time series | Record restrictions on internal movement between cities/regions |
| C8_International travel controls* | OxCGRT | Ordinal time series | Record restrictions on international travel |
| H1_Public information campaigns* | OxCGRT | Ordinal time series | Record presence of public info campaigns |
| H2_Testing policy* | OxCGRT | Ordinal time series | Record government policy on who has access to PCR testing |
| H3_Contact tracing* | OxCGRT | Ordinal time series | Record government policy on contact tracing after a positive diagnosis |
| H6_Facial Coverings* | OxCGRT | Ordinal time series | Record policies on the use of facial coverings outside the home |
| StringencyIndex* | OxCGRT | Time series | Record the strictness of “lockdown style” policies that primarily restrict people’s behavior |
| ContainmentHealthIndex* | OxCGRT | Time series | Combines ‘lockdown’ restrictions and closures with measures such as testing policy and contact tracing, short term investment in healthcare, as well investments in vaccine |
| GHS_score | ghsindex.org | Time invariant control | Benchmarking of health security and related capabilities |
| prevent_score | ghsindex.org | Time invariant control | Record ability to prevent the emergence or release of pathogens |
| detect_score | ghsindex.org | Time invariant control | Record for detection capability |
| GDPP (thousands) | IMF WEO | Time invariant control | GDP at constant price purchasing-power parity |
| CO2_emission (Mkt) | World Bank | Time invariant control | Carbon dioxide emissions stemming from the burning of fossil fuels and the manufacture of cement. |
| tot_greenhouse_MktCO2 | World Bank | Time invariant control | Total greenhouse gas emissions in kt of CO_2_ equivalent |
| tot_methane_MktCO2 | World Bank | Time invariant control | Methane emissions stemming from human activities in kt of CO_2_ equivalent |
| tot_NOX_MktCO2 | World Bank | Time invariant control | Nitrous oxide emissions from agricultural biomass burning, industrial activities, and livestock management activities in kt of CO_2_ |
| PM2.5_year_exposure_mcg/m3 | World Bank | Time invariant control | Population-weighted exposure to ambient PM2.5 pollution |
| pop_density | World Bank and UNDESA | Time invariant control | Population density |
| mobile_sub_100pp | World Bank | Time invariant control | Mobile cellular subscriptions (per 100 people) |
| internet_servers_1Mpp | World Bank | Time invariant control | Secure Internet servers (per 1 million people) |
| pop_age_65more (%) | World Bank | Time invariant control | Total population 65 years of age or older (as % of total population) |
| female_pop (%) | World Bank | Time invariant control | Total female population (as % of total population) |
| urban_pop (%) | World Bank | Time invariant control | People living in urban areas (as % of total population) |

See OxCGRT Codebook <https://github.com/OxCGRT/covid-policy-tracker/blob/master/documentation/codebook.md> or the index methodology documentation: <https://github.com/OxCGRT/covid-policy-tracker/blob/master/documentation/index_methodology.md> for more information on the definitions for each variable [51].
