# Supplementary material for: Does climate help modeling COVID-19 risk and to what extent?
Source: PLoS One. 2022 Sep 7;17(9):e0273078. doi: 10.1371/journal.pone.0273078 (PMC9451080; doi:10.1371/journal.pone.0273078)
Supplement: S3 Table — (DOCX) [file pone.0273078.s009.docx]

**S3 Table. Descriptive statistics**

| **Variable name** | **count** | **mean** | **std** | **min** | **25%** | **50%** | **75%** | **max** |
| --- | --- | --- | --- | --- | --- | --- | --- | --- |
| daily_cases | 77,283 | 1,871.96 | 8,644.45 | 11.00 | 49.00 | 230.00 | 981.00 | 299,786.00 |
| days_from_start | 76,892 | 177.03 | 111.11 | 0.00 | 73.00 | 172.00 | 274.00 | 413.00 |
| Temperature | 77,283 | 15.75 | 10.31 | -33.56 | 8.46 | 17.06 | 24.60 | 42.80 |
| Absolute humidity | 77,283 | 10.33 | 5.64 | 0.22 | 5.74 | 9.04 | 14.52 | 27.85 |
| Pressure | 77,283 | 968.64 | 50.79 | 727.78 | 956.44 | 986.48 | 1,001.15 | 1,042.53 |
| Wind speed | 77,283 | 3.16 | 1.72 | 0.08 | 1.93 | 2.82 | 4.01 | 14.32 |
| Rainfall | 77,283 | 3.09 | 6.77 | 0.00 | 0.03 | 0.55 | 3.21 | 200.66 |
| Short-wave irradiation | 77,283 | 4,847.37 | 2,000.20 | 9.53 | 3,436.39 | 5,005.67 | 6,339.85 | 9,832.04 |
| PM2P5 | 77,283 | 12.82 | 13.65 | 0.01 | 6.01 | 9.26 | 14.98 | 829.33 |
| PM10 | 77,283 | 25.42 | 29.37 | 0.02 | 10.19 | 16.12 | 27.82 | 1,187.67 |
| UV | 77,283 | 1.89 | 0.78 | 0.01 | 1.33 | 1.98 | 2.48 | 3.90 |
| C1_School closing* | 74,066 | 2.33 | 0.83 | 0.00 | 2.00 | 3.00 | 3.00 | 3.00 |
| C2_Workplace closing* | 73,746 | 1.86 | 0.82 | 0.00 | 1.00 | 2.00 | 2.00 | 3.00 |
| C3_Cancel public events* | 74,075 | 1.71 | 0.53 | 0.00 | 1.00 | 2.00 | 2.00 | 2.00 |
| C4_Restrictions on gatherings* | 74,066 | 3.16 | 1.18 | 0.00 | 3.00 | 4.00 | 4.00 | 4.00 |
| C5_Close public transport* | 74,071 | 0.72 | 0.71 | 0.00 | 0.00 | 1.00 | 1.00 | 2.00 |
| C6_Stay at home requirements* | 74,067 | 1.37 | 0.84 | 0.00 | 1.00 | 1.00 | 2.00 | 3.00 |
| C7_Restrictions on internal movement* | 74,083 | 1.24 | 0.78 | 0.00 | 1.00 | 1.00 | 2.00 | 2.00 |
| C8_International travel controls* | 73,888 | 2.96 | 0.92 | 0.00 | 3.00 | 3.00 | 4.00 | 4.00 |
| H1_Public information campaigns* | 74,072 | 1.97 | 0.18 | 0.00 | 2.00 | 2.00 | 2.00 | 2.00 |
| H2_Testing policy* | 74,059 | 2.03 | 0.80 | 0.00 | 1.00 | 2.00 | 3.00 | 3.00 |
| H3_Contact tracing* | 73,986 | 1.51 | 0.58 | 0.00 | 1.00 | 2.00 | 2.00 | 2.00 |
| H6_Facial Coverings* | 73,633 | 2.29 | 1.34 | 0.00 | 1.00 | 3.00 | 3.00 | 4.00 |
| StringencyIndex | 74,065 | 65.42 | 16.79 | 0.00 | 54.17 | 65.74 | 78.70 | 100.00 |
| ContainmentHealthIndex | 74,059 | 60.88 | 12.18 | 0.00 | 53.85 | 61.54 | 69.87 | 91.35 |
| GHS_score | 77,004 | 57.34 | 19.51 | 16.20 | 42.10 | 55.40 | 75.60 | 83.50 |
| prevent_score | 77,004 | 54.10 | 21.62 | 1.90 | 36.50 | 50.90 | 81.10 | 83.10 |
| detect_score | 77,004 | 65.34 | 27.60 | 2.70 | 41.70 | 71.20 | 96.40 | 98.20 |
| GDPP (thousands) | 77,283 | 31.09 | 27.01 | 0.31 | 5.75 | 23.33 | 62.87 | 185.74 |
| CO2_emission (Mkt) | 76,634 | 1.56 | 2.35 | 0.00 | 0.03 | 0.21 | 2.24 | 10.29 |
| tot_greenhouse_MktCO2 | 75,247 | 2.02 | 2.83 | 0.00 | 0.06 | 0.38 | 6.34 | 12.45 |
| tot_methane_MktCO2 | 76,458 | 0.20 | 0.28 | 0.00 | 0.01 | 0.05 | 0.50 | 1.75 |
| tot_NOX_MktCO2 | 76,639 | 0.09 | 0.13 | 0.00 | 0.00 | 0.02 | 0.24 | 0.59 |
| PM2.5_year_exposure_mcg/m3 | 76,754 | 21.04 | 19.03 | 5.86 | 7.41 | 15.02 | 24.79 | 99.73 |
| pop_density | 76,934 | 251.30 | 1,082.72 | 0.02 | 25.70 | 76.28 | 167.20 | 26,338.30 |
| mobile_sub_100pp | 77,276 | 117.75 | 26.93 | 20.36 | 103.17 | 126.83 | 129.01 | 208.50 |
| internet_servers_1Mpp | 77,012 | 26,075.51 | 30,642.62 | 0.31 | 329.30 | 11,670.80 | 65,767.56 | 264,256.63 |
| pop_age_65more (%) | 76,573 | 0.50 | 0.04 | 0.26 | 0.50 | 0.51 | 0.52 | 0.68 |
| female_pop (%) | 76,573 | 0.64 | 0.21 | 0.13 | 0.51 | 0.67 | 0.81 | 1.04 |
| urban_pop (%) | 77,012 | 0.10 | 0.07 | 0.01 | 0.04 | 0.07 | 0.16 | 0.28 |
